# Macrocyclic and linear peptides promote LAR dimerization and neurite outgrowth

**DOI:** 10.1016/j.jbc.2026.113340

**Published:** 2026-07-16

**Authors:** Misato Yasumura, Yuchen Zhang, Tokuichi Iguchi, Naoakusa Fujimura, Kilian Colas, Yuichiro Oka, Hiroyuki Oshikane, Tsuyoshi Inoue, Hiroaki Suga, Makoto Sato

**Affiliations:** 1 Department of Anatomy and Neuroscience, Graduate School of Medicine, The University of Osaka, Osaka, Japan; 2 Department of Chemistry, Graduate School of Science, The University of Tokyo, Tokyo, Japan; 3 United Graduate School of Child Development, Osaka University, Kanazawa University, Hamamatsu University School of Medicine, Chiba University, and University of Fukui (UGSCD), The University of Osaka, Osaka, Japan; 4 Division of Advanced Pharmaco-Science, Graduate School of Pharmaceutical Science, The University of Osaka, Osaka, Japan

**Keywords:** cyclic peptide, dimerization, tyrosine-protein phosphatase, tyrosine phosphatase, high-throughput screening, HTS, neurite outgrowth

## Abstract

Leucocyte common antigen-related protein (LAR), a member of the type IIa receptor protein tyrosine phosphatase subfamily, regulates biological processes such as cell differentiation, migration, axon elongation, and axon regeneration through its phosphatase activity or cell–cell interactions. The phosphatase activity of LAR is negatively regulated by its dimerization driven by the interaction with extracellular or cytoplasmic molecules. In the nervous system, monomeric LAR suppresses axon elongation and regeneration, while dimeric or oligomeric LAR exhibits the opposite effects. However, intracellular signaling mechanisms associated with LAR dimerization remain incompletely understood due to the lack of tools to control LAR dimerization at will. In this study, we performed peptide library screening using the random nonstandard peptides integrated discovery system, which combines mRNA display with genetic code reprogramming. Through this approach, we identified high-affinity LAR-binding macrocyclic and linear peptides, named L6 and D1L, respectively. These peptides promoted the dimerization of both mouse and human LAR with minimal off-target effects in a split luciferase assay. When L6 or D1L was dimerized using a cross-linker, both peptides showed enhanced LAR dimerization activity, with D1L-derived dimers exhibiting the strongest effect. Furthermore, both monomeric and dimeric peptides promoted neurite outgrowth in cultured hippocampal neurons. Our results identify the *de novo* peptides as selective modulators of LAR dimerization, providing new tools to probe LAR-mediated signaling in axon growth and regeneration.

Leukocyte common antigen-related protein (LAR) belongs to receptor protein tyrosine phosphatases (RPTPs) and is a member of the type IIa RPTP subfamily, also known as LAR-RPTP subfamily (1). This subfamily includes three vertebrate homologs: LAR, PTPδ, and PTPσ. LAR-RPTPs share a common domain structure consisting of a large extracellular domain, a single-pass transmembrane region, tandem cytoplasmic PTP domains (D1 and D2) (Fig. 1*A*) (2). The membrane-proximal D1 domain is catalytically active, while the membrane-distal D2 domain lacks phosphatase activity (3). The extracellular domain includes three immunoglobulin (Ig)-like domains and four to eight fibronectin type III (FN) domains (2). LAR-RPTPs have multiple variants generated through alternative splicing, involving the entire extracellular region (such as Ig3, FN5, or FN4-7) and four micro-exon inserts (meA, meB, meC, and meD) (4). The expression of these variants is tissue-specific throughout development (4).

**Figure 1:**
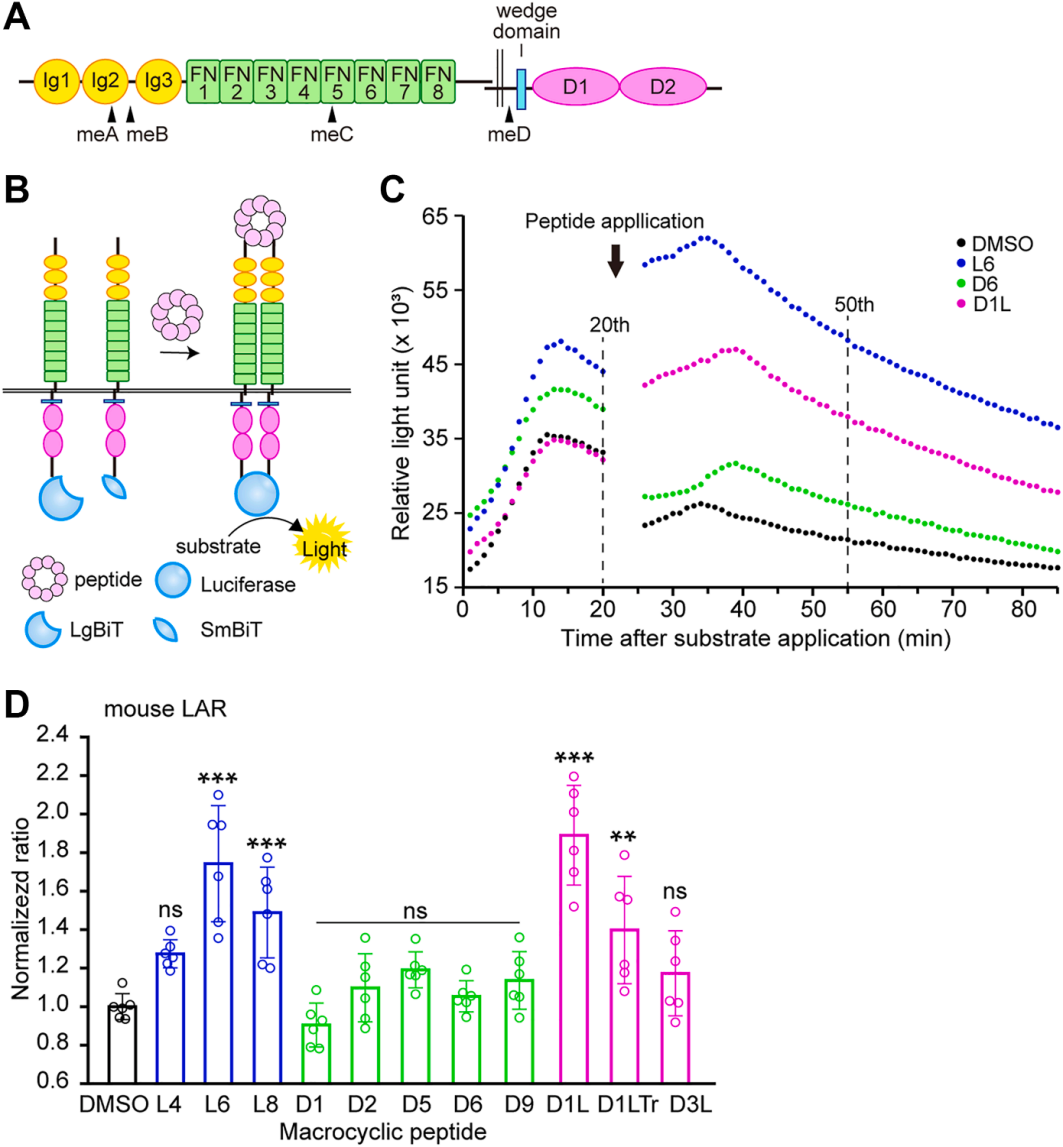
**Macrocyclic peptides promote mouse LAR dimerization.***A*, schematic drawing of the domain structure of LAR. Domains and splice variants related to micro-exon inserts are shown. *B*, schematic drawing of the split luciferase assay. LgBiT or SmBiT were fused with the C terminus of LAR. When a peptide induces LAR dimerization, luciferase activity is reconstituted, which can be measured quantitatively after adding a substrate. *C*, examples of luminescence changes in a split luciferase assay. Luminescence was measured for 20 min before and 60 min after macrocyclic peptide application. The 50th/20th measurement ratio was calculated as an index of LAR dimerization activity and normalized to the DMSO-treated control. The data represent the results of quadruplicate analyses. *D*, effects of 10 μM macrocyclic peptide application on mouse LAR (mLAR) dimerization in HEK293T cells coexpressing mLAR-LgBiT and mLAR-SmBiT. Cyclic L6 (1.74 ± 0.30; *p* < 0.0001), L8 (1.49 ± 0.24; *p* = 0.0004), linear D1L (1.89 ± 0.26; *p* < 0.0001), and D1LTr (1.40 ± 0.28; *p* = 0.0053) significantly promoted mLAR dimerization compared to the DMSO-treated control. On the other hand, cyclic L4 (1.27 ± 0.07; *p* = 0.11), D1 (0.90 ± 0.11; *p* = 0.97), D2 (1.10 ± 0.18; *p* = 0.97), D5 (1.19 ± 0.09; *p* = 0.45), D6 (1.05 ± 0.08; *p* = 1.00), D9 (1.14 ± 0.15; *p* = 0.81), and linear D3L (1.17 ± 0.22; *p* = 0.57) did not promote mLAR dimerization. Data are represented as mean ± SD. Individual data points are shown as *open circles*. n = 6 independent experiments. Each experiment was performed in quadruplicate. ∗∗∗*p* < 0.001, ∗∗*p* < 0.01, ns: not significant; Dunnett’s test. D1 and D2, phosphatase domains; DMSO, dimethyl sulfoxide; FN, fibronectin type III domain; HEK, human embryonic kidney; Ig, immunoglobulin-like domain; LAR, leukocyte common antigen-related protein; LgBiT, large fragment of NanoBiT; SmBiT, small fragment of NanoBiT.

LAR regulates various cellular processes, including cytoskeletal organization, cell differentiation, adhesion, and migration by modulating the phosphorylation state of its substrates (5, 6, 7, 8). In the nervous system, it functions as a cell adhesion molecule involved in multiple events, such as neurite outgrowth in cultured neurons, axon guidance and regeneration, and synapse formation in both peripheral and central neurons (9, 10, 11, 12, 13, 14, 15). Notably, early *in vivo* evidence from *Caenorhabditis elegans* demonstrated that the LAR homolog PTP-3 plays a crucial role in axon development (16). In addition, PTP-3 has also been shown to regulate neuroblast migration (17), suggesting that the functions of LAR in axon development and cell migration are evolutionarily conserved.

LAR mediates cell–cell or cell–extracellular matrix interactions to regulate axon development, often by binding to chondroitin sulfate proteoglycans (CSPGs) and heparan sulfate proteoglycans (HSPGs) (5). Interactions between LAR-RPTPs and HSPGs promote axon outgrowth, whereas interactions with CSPGs exert the inhibitory effect (18). CSPGs promote LAR monomerization and enhance its phosphatase activity, leading to the suppression of axon growth. This inhibitory effect is associated with the downregulation of crucial signaling pathways involved in axon growth, including the inactivation of Akt, Erk, and PKCζ, and the activation of RhoA signaling (4, 9, 19). Despite the previously accumulated knowledge, the molecular mechanisms by which CSPG–LAR interactions regulate these pathways remain unclear (19). Interestingly, treatment with a small peptide corresponding to the fifth FN domain of LAR (LARFN5C), which blocks LAR homophilic interactions by binding to its ectodomain, has been shown to enhance neurite extension and nerve regeneration (9, 20). These promotive effects are attributed to the activation of protein tyrosine kinases such as Src, FAK, and TrkB, which subsequently activate Akt, Erk, and PKC (20). The mechanisms underlying this contrasting effect on axon development, compared to CSPG–LAR interaction, are also not yet fully understood. Unlike CSPGs, the effects of LAR-RPTPs interacting with HSPGs on promoting axon growth in the mammalian nervous system have been primarily studied in the context of PTPσ (18, 21, 22). HSPGs promote PTPσ dimerization or oligomerization while suppressing its phosphatase activity. LAR binds to HSPGs such as glypican and syndecan (23); however, how dimerized or oligomerized LAR regulates intracellular signaling related to axon growth remains to be fully elucidated. To clarify the mechanisms by which monomerized, dimerized, or oligomerized LAR influences axon growth, tools are needed that can selectively regulate LAR clustering states. However, since vertebrate LAR, PTPδ, and PTPσ share overall amino acid sequences (∼70% identity and ∼80% similarity), and both CSPGs and HSPGs bind to the first Ig domain of LAR-RPTPs (18, 24), only LARFN5C and L59, a C-terminal fragment of LARFN5C, are reported as inhibitors of LAR dimerization (14, 20).

The random nonstandard peptides integrated discovery (RaPID) system utilizes mRNA display and genetic code reprogramming in the flexible *in vitro* translation system (25, 26). This approach enables the ribosomal synthesis of large libraries of natural product-like macrocyclic peptides and allows rapid selection of those with high affinity and selectivity for target proteins (25, 26). Macrocyclic peptides have gained notable interest as a new therapeutic class, providing an alternative to low-molecular-weight compounds and large biomolecules like antibodies. They have the advantages of large quantities through chemical synthesis, are easily modified, and remain stable under physiological reducing conditions (27, 28). In this study, we discovered high-affinity *de novo* macrocyclic and linear peptides targeting LAR Ig domains using the RaPID system. These peptides could bind to mouse and human LAR but not to mouse PTPδ and PTPσ. In addition, these peptides exhibited activity in promoting LAR dimerization and promoted neurite elongation in cultured hippocampal neurons.

## Results

### Identification of macrocyclic peptides against LAR Ig domains

The RaPID system was used to select peptides that bind to extracellular Ig-like domains of mouse LAR (referred to as mLAR-Ig). A library containing over 10^12^ unique macrocyclic peptides was applied to streptavidin-conjugated magnetic beads, which had C-terminally biotinylated mLAR-Ig attached to them for enriching peptides that target LAR. After eight rounds of selection, the recovery rate (Fig. S1, measured as the amount of DNA recovered from the input) of the library binding to the mLAR-Ig-immobilized beads had plateaued. Sequencing the recovered library identified eleven peptides: three (L4, L6, and L8) from the L-amino acid-initiated library and eight (D1, D2, D5, D6, D9, D1L, D1LTr, and D3L) from the D-amino acid-initiated library (Table 1). D1L, D1LTr, and D3L were linear peptides, which were not cyclized due to the absence of the C-terminal Cys caused by a frameshift. To confirm the binding ability of these peptides to LAR, we chemically synthesized these peptides and conducted surface plasmon resonance (SPR) assays. Identified peptides were injected over the LAR-Ig-tethered chip surface at a range of different concentrations. The sensorgrams depicting interactions of macrocyclic peptides with mLAR-Ig represented typical SPR binding responses. We analyzed the association and dissociation phases of these sensorgrams using a two-state reaction model. All peptides exhibited dissociation constants (*K*_D_) in the nanomolar range (1.8–380 nM) (Table 1 and Fig. S2).

**Table 1:** Sequences and kinetic constants of the selected macrocyclic peptides

Peptide name	Sequence^a^	*K*_D_ (nM)	*k*_a_ (10^6^ M^−1^s^−1^)	*k*_d_ (10^−2^ s^−1^)
L4	-S-Ac-YIGNHFHPGTRYS**C**-NH_2_	229	0.0644	1.48
L6	-S-Ac-YNLFFKHVHVYHR**C**-NH_2_	1.8	1.00	0.176
L8	-S-Ac-YSVEFAELSSYVLFS**C**-NH_2_	163	0.038	6.20
D1	-S-Ac-DYLITRYALVKTS**C**-NH_2_	91	0.0091	0.0888
D2	-S-Ac-DYIYLIKKNFKVYQAV**C**-NH_2_	40.3	0.245	0.987
D5	-S-Ac-DYSLFDASGHYIANYS**C**-NH_2_	145	0.0512	0.742
D6	-S-Ac-DYDLYNIKGQYIATYK**C**-NH_2_	15.8	1.65	2.06
D9	-S-Ac-DYSTKRLLLYEFVTVV**C**-NH_2_	144	0.224	3.23
D1L	ClAc-DYEYLISSRVYDELLRQRQRQLGRGA-NH_2_	5.9	0.168	0.0993
D1L-Tr	ClAc-DYEYLISSRVYDELL-NH_2_	96.2	0.0148	0.142
D3L	ClAc-DYTYLISAEAYSDLLRQRQRQLGRGAE-NH_2_	379	38.5	1360

Sequences of LAR-Ig-binding macrocyclic peptides obtained from the RaPID selection. The boldfaced letter indicates the Cys residue that is cyclized with the N-terminal ClAc moiety.

a The N-terminal thioether-acyl moiety (-S-Ac-) forms a thioether macrocycle with the side chain of cysteine residue in bold font (the actual macrocyclic chemical structure of L6 is shown in Fig. 3*A*). D1L, D1L-Tr, and D3L were linear peptides with *N*-terminal chloroacetyl moiety (ClAc), and others were thioether macrocycles. The dissociation (*K*_D_), association rate (*k*_a_), and dissociation rate (*k*_d_) constants were determined by surface plasmon resonance using immobilized mouse LAR extracellular Ig-like domain.

### Macrocyclic and linear peptides dimerized mLAR in HEK293T cells

Next, we examined whether the identified peptides could dimerize mLAR using a split luciferase assay (NanoLuc Binary Technology; NanoBiT) (29). In this system, either a large (LgBiT) or a small (SmBiT) fragment of NanoBiT was fused at the C terminus of LAR. When peptides induce dimerization of LAR, the NanoBiT fragments come into proximity, leading to luciferase activity reconstitution, which can be measured quantitatively after adding a substrate (Fig. 1*B*). We cotransfected HEK293T cells with expression vectors encoding mLAR fused to LgBiT and SmBiT at the C terminus. Since dimeric LAR exists in living cells (30), an increase in luminescence was observed upon substrate addition (Fig. 1*C*). We evaluated the ability of the peptides to dimerize mLAR by comparing luminescence before and after application. The luminescence at the 50th measurement was divided by that at the 20th measurement, and this ratio was used as an index of LAR dimerization. All values were normalized to the dimethyl sulfoxide; DMSO-treated control. When applying L4, D1, D2, D5, D6, D9, or D3L to HEK293T cells, the ratios of luminescence changes were similar to those with DMSO treatment. Conversely, applying L6, L8, D1L, or D1LTr significantly increased the ratios of luminescence changes compared to the DMSO-treated control (Fig. 1*D*), suggesting that these peptides can dimerize mLAR by binding to it. The extent of dimerization varied depending on the peptide used. Notably, L6 and D1L peptides exhibited robust dimerization activity toward mLAR, consistent with their high binding affinity for LAR (Table 1).

### L6 and D1L dimerized both mLAR and hLAR

**Figure 2:**
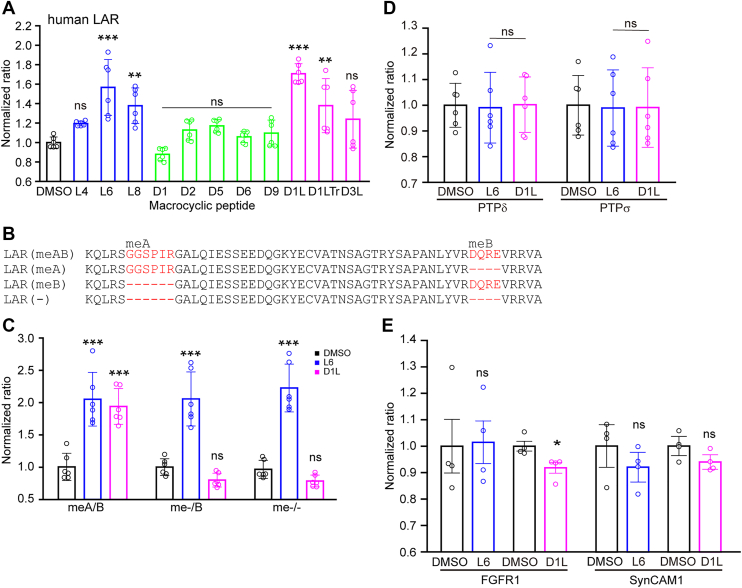
**Macrocyclic peptides selectively promote mouse and human LAR dimerization but do not promote mouse PTPδ and PTPσ dimerization.***A*, effects of 10 μM macrocyclic peptide on human LAR (hLAR) dimerization in HEK293T cells coexpressing hLAR-LgBiT and hLAR-SmBiT. Cyclic L6 (1.57 ± 0.29; *p* < 0.0001), L8 (1.38 ± 0.18; *p* = 0.0022), linear D1L (1.71 ± 0.10; *p* < 0.0001), and D1LTr (1.38 ± 0.28; *p* = 0.0022) significantly promoted hLAR dimerization compared to the DMSO-treated control. On the other hand, Cyclic L4 (1.19 ± 0.02; *p* = 0.29), D1 (0.88 ± 0.06; *p* = 0.80), D2 (1.13 ± 0.10; *p* = 0.75), D5 (1.17 ± 0.06; *p* = 0.43), D6 (1.06 ± 0.06; *p* = 1.00), D9 (1.10 ± 0.13; *p* = 0.94), and linear D3L (1.24 ± 0.30; *p* = 0.12) did not promote hLAR dimerization. n = 6 independent experiments. Each experiment was performed in triplicate or quadruplicate. *B*, amino acid sequences surrounding the micro-exon (meA and meB) of mouse LAR splice variants. *C*, effects of 10 μM L6 or D1L on dimerization of mLAR(meA/B), mLAR(me−/B), or mLAR(me−/−) in HEK293T cells coexpressing the corresponding LgBiT and SmBiT fusion constructs. L6 significantly promoted dimerization of all mLAR variants: mLAR(meA/B) (2.04 ± 0.41; *p* < 0.0001), mLAR(me−/B) (2.05 ± 0.42; *p* < 0.0001), and mLAR(me−/−) (2.30 ± 0.38; *p* < 0.0001). In contrast, D1L significantly promoted dimerization only of mLAR(meA/B) (1.93 ± 0.27; *p* = 0.0002), but not of mLAR(me−/B) (0.80 ± 0.11; *p* = 0.33) and mLAR(me−/−) (0.81 ± 0.09; *p* = 0.34). n = 6 independent experiments. Each experiment was performed in duplicate or triplicate. *D*, effects of 10 μM L6 or D1L on dimerization of mouse PTPδ or PTPσ in HEK293T cells coexpressing PTPδ-LgBiT and -SmBiT or PTPσ-LgBiT and -SmBiT. Neither L6 nor D1L promoted dimerization of PTPδ or PTPσ. L6 (0.99 ± 0.14; *p* = 0.98) and D1L (1.00 ± 0.11; *p* = 1.00) for PTPδ and L6 (0.99 ± 0.15; *p* = 0.99) and D1L (0.99 ± 0.15; *p* = 0.99) for PTPσ. n = 6 independent experiments. Each experiment was performed in quadruplicate. *E*, effects of 10 μM L6 or D1L on dimerization of mouse FGFR1 or SynCAM1 in HEK293T cells coexpressing FGFR1-LgBiT and -SmBiT or SynCAM1-LgBiT and -SmBiT. Neither L6 (0.92 ± 0.07; *p* = 0.27) nor D1L (0.94 ± 0.04; *p* = 0.09) promoted dimerization of SynCAM1. In contrast, D1L (0.92 ± 0.04; *p* = 0.026) but not L6 (1.01 ± 0.16; *p* = 0.91) inhibited FGFR1 dimerization. n = 4 independent experiments. Each experiment was performed in triplicate or quadruplicate. All data are represented as mean ± SD. Individual data points are shown as *open circles*. ∗∗∗*p* < 0.001, ∗∗*p* < 0.01, ∗*p* < 0.05, ns: not significant; Dunnett’s test (*A*, *C*, and *D*) or unpaired *t* test (*E*). DMSO, dimethyl sulfoxide; FGFR1, fibroblast growth factor receptor 1; HEK, human embryonic kidney; LAR, leukocyte common antigen-related protein; LgBiT, large fragment of NanoBiT; SmBiT, small fragment of NanoBiT; SynCAM1, synaptic cell adhetion molecule 1.

**Figure 3:**
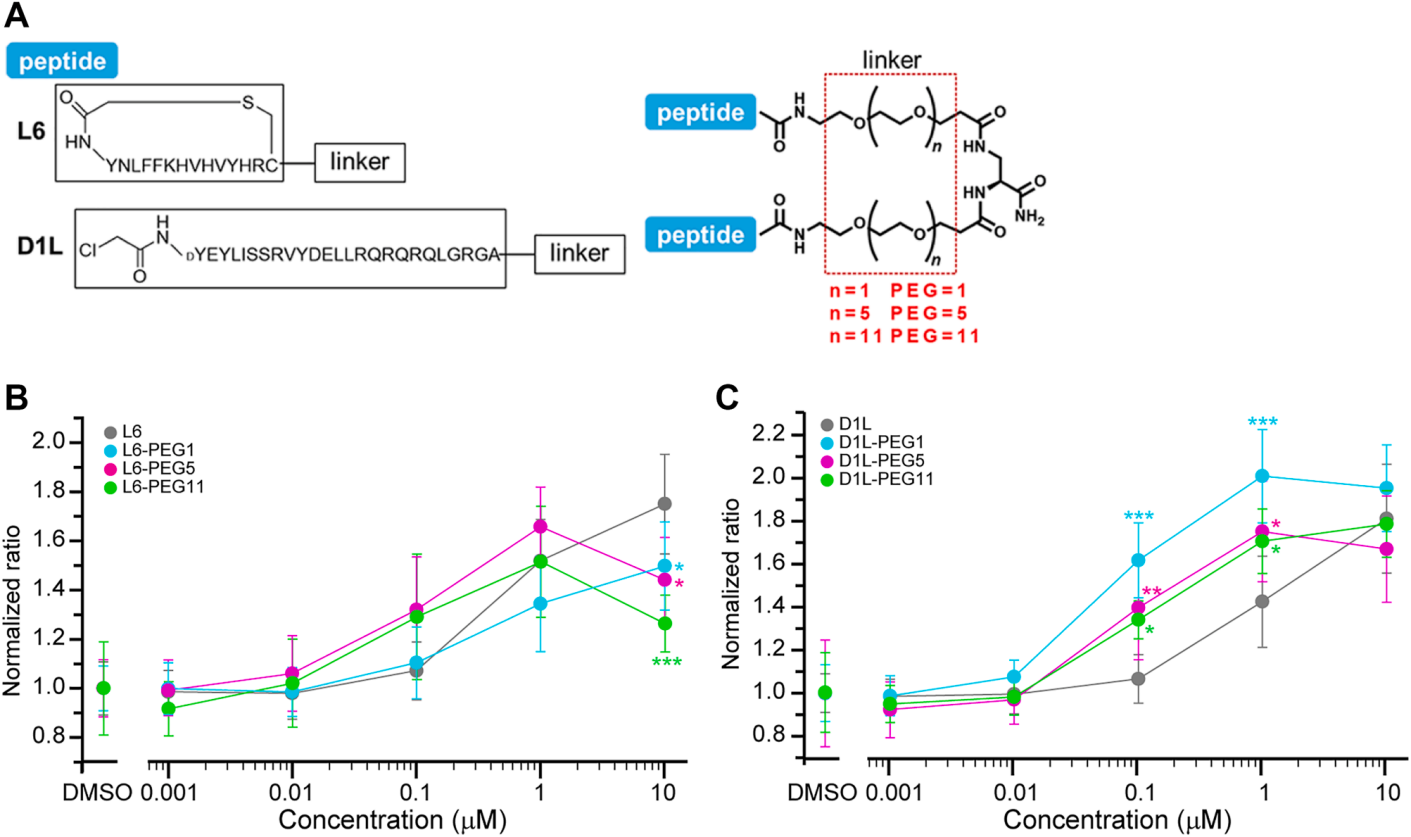
**Dimeric L6 and D1L promote mLAR dimerization more effectively than monomeric L6 or D1L.***A*, structure of dimeric L6 and D1L. Monomeric peptides were dimerized using Fmoc-NH-PEGX-COOH linker. *B* and *C*, effects of dimeric L6 (*B*) and D1L (*C*) on mLAR dimerization in HEK293T cells coexpressing mLAR-LgBiT and mLAR-SmBiT. L6-PEG5 (1.32 ± 0.22; *p* = 0.093) and L6-PEG11 (1.29 ± 0.25; *p* = 0.15) showed a tendency to promote mLAR dimerization compared to monomeric L6 (1.07 ± 0.12) at 100 nM. D1L-PEG1 (100 nM: 1.66 ± 0.19; *p* < 0.0001, 1 μM: 2.07 ± 0.23; *p* = 0.0001), D1L-PEG5 (100 nM: 1.42 ± 0.26; *p* = 0.0054, 1 μM: 1.80 ± 0.25; *p* = 0.018), and D1L-PEG11 (100 nM: 1.36 ± 0.09; *p* = 0.022, 1 μM: 1.75 ± 0.16; *p* = 0.046) significantly promoted mLAR dimerization compared to monomeric D1L at 100 nM (1.07 ± 0.11) or 1 μM (1.43 ± 0.21). Data are represented as mean ± SD. n = 6 independent experiments. Each experiment was performed in quadruplicate. Statistical significance was determined using two-way repeated measures ANOVA (peptide type x concentration) followed by Dunnett’s test. ∗∗∗*p* < 0.001, ∗∗*p* < 0.01, ∗*p* < 0.05. HEK, human embryonic kidney; LgBiT, large fragment of NanoBiT; SmBiT, small fragment of NanoBiT.

Ig-like domains of mouse and human LAR share sequence similarities. We asked whether identified peptides could promote hLAR dimerization as they did with mLAR. The mLAR constructs used in this study include meA, C, and D, and lack FN4, 6, and 7. Therefore, we used hLAR constructs with the same domain structures in a split luciferase assay. Application of L6, L8, D1L, or D1LTr but not L4, D1, D2, D5, D6, D9, or D3L to hLAR-LgBiT- and hLAR-SmBiT- (referred to as hLAR-LgBiT/SmBiT) expressing HEK293T cells significantly increased the ratios of luminescence changes compared to DMSO treatment (Fig. 2*A*). The ratio values for each peptide were similar to those observed with mLAR. Thus, these peptides can induce dimerization of both mouse and human LAR. The N terminus of linear peptide D1L was chloroacetylated (Fig. 3*A*), allowing potential covalent interactions with nucleophilic amino acid residues such as cysteine, serine, lysine, and histidine. To assess the contribution of covalent binding, we examined whether N-terminally acetylated D1L (Ac-D1L; Fig. S3*A*) could induce LAR dimerization. Unlike D1L, Ac-D1L lacks the reactive chloroacetyl moiety and therefore is not expected to form covalent interactions with nucleophilic amino acid residues. Application of Ac-D1L showed a tendency to enhance LAR dimerization; however, this effect did not reach statistical significance (Fig. S3*B*), suggesting that covalent bonding strengthens the interaction between LAR and D1L. Considering the strong dimerization ability of the L6 and D1L peptides, we focused on these two for further analyses.

Four LAR isoforms carrying meA and meB in Ig-like domains exist in the mouse brain (10, 31, 32). We investigated whether L6 and D1L can dimerize all mLAR variants using a split luciferase assay (Fig. 2*B*). Applying the macrocyclic peptide L6 to HEK293T cells expressing mLAR(meAB)-, mLAR(meB)-, and mLAR(−)-LgBiT/SmBiT, as well as mLAR(meA)-LgBiT/SmBiT expressing HEK293T cells, significantly increased the ratios of luminescence changes compared to DMSO-treated control (Figs. 1*D* and 2*C*). On the other hand, applying the linear peptide D1L to HEK293T cells expressing mLAR(meB)-LgBiT/SmBiT or mLAR(−)-LgBiT/SmBiT did not increase the ratios of luminescence changes (Fig. 2*C*). In contrast, treatment with D1L significantly increased the ratios of luminescence changes in HEK293T cells expressing mLAR(meAB)-and mLAR(meA)-LgBiT/SmBiT compared to DMSO-treated control (Figs. 1*D* and 2*C*). These results suggest that L6 dimerized mLAR in an meA- and meB-independent manner, and D1L did so in an meA-dependent manner. To gain structural insight into these binding differences, we performed computational modeling using ColabFold (AlphaFold2-based prediction), HADDOCK, and Rosetta (Fig. S4) (33, 34, 35). These analyses consistently predicted that D1L binds near the meA insertion region, providing a possible structural explanation for its selective activity towards meA-containing LAR isoforms, whereas L6 was predicted to bind to the third Ig-like domain, consistent with its activity across all splice variants. These predictions are consistent with the biochemical results showing distinct splice variant specificity. The predicted interfaces within the Ig-like domain, however, vary across prediction methods.

The LAR-RPTPs have similar domain structures, and their overall amino acid sequence similarities or identities are high (24). To confirm the specific binding of the peptides to LAR, we repeated the experiments with mouse PTPσ(meB) or PTPδ(meAB). As shown in Figure 1*D*, applying L6 or D1L to mLAR-LgBiT/SmBiT-expressing HEK293T cells strongly promoted LAR dimerization, while the increases in the ratios of luminescence changes were not observed in PTPσ(meB) or PTPδ(meAB)-LgBiT/SmBiT-expressing HEK293T cells (Fig. 2*D*). To further evaluate potential off-target effects, we examined whether the peptides promote dimerization of mouse fibroblast growth factor receptor 1 (FGFR1) and synaptic cell adhesion molecule 1 (SynCAM1), both of which are Ig-like domain-containing cell-surface receptors. L6 did not affect luminescence ratios in HEK293T cells expressing FGFR1-or SynCAM1-LgBiT/SmBiT (Fig. 2*E*). In contrast, D1L modestly decreased the luminescence ratio in HEK293T cells expressing either FGFR1-or SynCAM1-LgBiT/SmBiT (Fig. 2*E*). This effect was not observed with Ac-D1L (Fig. S3*C*), suggesting that it is mediated by a covalent interaction. Although only a limited number of receptors were tested, these results suggest that the peptides preferentially promote LAR dimerization over other tested Ig-like cell-surface receptors. Peptide treatment did not reduce cell viability or induce morphological changes (Fig. S5, *A* and *B*). In addition, no apparent aggregation was detected in SDS-PAGE at the concentrations used in the split luciferase assays (Fig. S5*C*). These findings suggest that the observed dimerization effects are unlikely to be driven by nonspecific peptide aggregation under the experimental conditions employed. Collectively, these data suggest that L6 and D1L targeting mLAR cross-reactively bind to both mLAR and hLAR and efficiently induce the dimerization of targets regardless of the origin, while exhibiting minimal off-target effects under the conditions tested.

### Dimeric L6 and D1L promoted LAR dimerization

A previous study reported that a dimeric Plexin B1-binding macrocyclic peptide showed a nearly 300-fold increase in binding affinity due to the elevated avidity, resulting in a significant improvement of biological activity compared to the monomeric counterpart (36). We examined whether dimeric L6 or D1L more effectively dimerizes LAR than the parental L6 or D1L using a split luciferase assay. We synthesized a series of L6 or D1L dimeric macrocycles using PEG1, PEG5, and PEG11 cross-linkers anchored to 2,3-diamino propanoic acid (Fig. 3*A*) (36). We transfected mLAR-LgBiT and mLAR-SmBiT in HEK293T cells and applied each dimerized peptide. Various concentrations in the range of 1 nM to 10 μM of monomeric L6 and D1L as well as their dimeric counterparts were tested for cellular activity by the above reporter system.

The avidity enhancement by dimerization of L6 was somewhat limited. At 100 nM concentration, L6-PEG5 and L6-PEG11 showed a tendency to elevate the ratio of luminescence change to 1.3 compared with the monomers, which gave a near background level of 1 (Fig. 3*B*). Interestingly, L6-PEG1, having the shortest linker, exhibited a similar range of activity to the monomeric L6. At 1 μM concentration, the activities of L6-PEG5 and L6-PEG11 were further elevated to 1.5 to 1.7, whereas L6-PEG1 showed even lower activity than L6 itself. At 10 μM concentration, the activities of dimeric peptides were decreased to a level of 1.3 to 1.5, whereas the monomeric L6 further increased to 1.8 (*vide infra* for more discussion). Interestingly, the dimeric D1L series behaved slightly differently from L6 (Fig. 3*C*). At 100 nM concentration, D1L-PEG1 bearing the shortest linker elevated the ratio of luminescence change to 1.7, and other dimeric D1L also showed an elevation to a 1.4 level, whereas D1L itself stayed at a near background level. At 1 μM concentration, D1L-PEG1 took off to nearly 2.1, and others were at a 1.7 to 1.8 level, whereas D1L increased only 1.4. Further increase in concentrations to 10 μM elevated all peptides in the range of 1.7 to 2.0. Overall, dimerization enhanced the activity of both L6 and D1L; however, the effect was considerably pronounced for D1L-derived dimers, particularly D1L-PEG1.

### Neurite elongation was enhanced by monomeric and dimeric peptides in cultured hippocampal neurons

**Figure 4:**
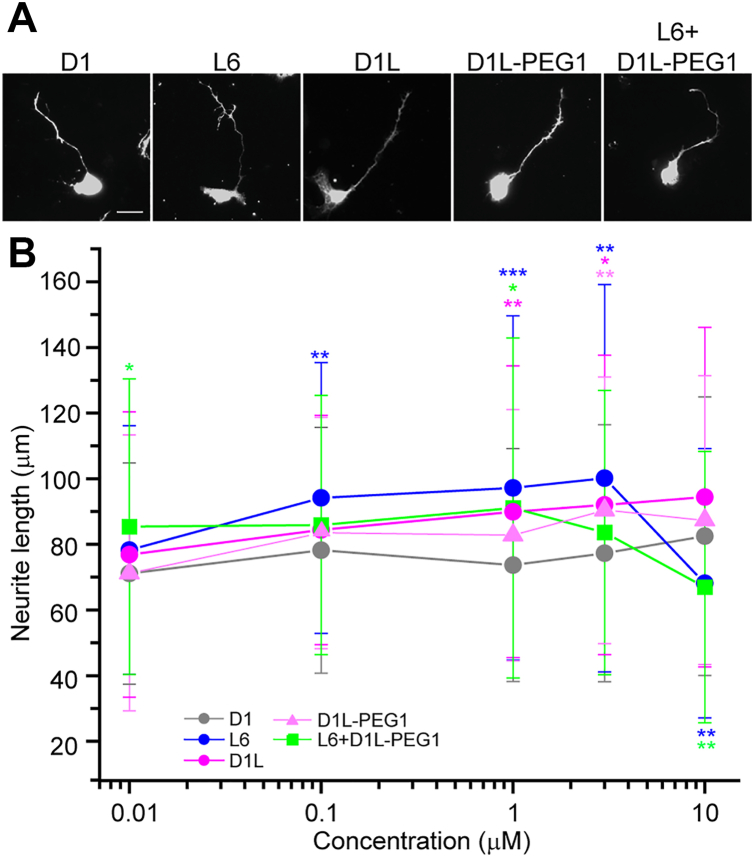
**Monomeric and dimeric peptides promote neurite elongation in cultured hippocampal neurons.***A*, representative images of cultured hippocampal neurons treated with 1 μM L6, D1L, D1L-PEG1, or a combination of L6 and D1L-PEG1 (L6 + D1L-PEG1). D1 was used as a control. Primary hippocampal cultures were prepared from *Fezf2*-EGFP or *Fezf2*-tdTomato mice. Peptides were added 3 h after plating at the indicated concentrations. After 48 h, neurons were fixed and immunostained with an anti-GFP or anti-RFP antibody. The scale bar represents 20 μm. *B*, quantification of neurite length. Isolated neurons were selected, and the length of the longest neurite was measured for each neuron. n = 135 to 152 neurons from 3 independent cultures. Data are represented as mean ± SD. Correlations between neurite length and peptide concentration were analyzed using a two-tailed Jonckheere-Terpstra trend test: D1L (*p* = 0.0010), D1L-PEG1 (*p* = 0.0002), and L6 + D1L-PEG1 (*p* = 0.0006) showed significant trends, whereas D1 (*p* = 0.074) and L6 (*p* = 0.14) did not. Statistical significance at each concentration was determined using Steel’s test. ∗∗∗*p* < 0.001, ∗∗*p* < 0.01, ∗*p* < 0.05. RFP, red fluorescent protein.

HS chains of HSPG induce LAR-RPTPs oligomerization, and LAR-RPTPs–HSPG interactions promote neuronal extension (18). We examined whether L6, D1L, or D1L-PEG1 peptides could induce functional dimerization of LAR and promote neurite elongation in cultured hippocampal neurons. Primary hippocampal neurons were prepared from *Fezf2*-EGFP or *Fezf2*-tdTomato mice, in which neurons were labeled with EGFP or tdTomato, respectively. Each peptide was added to the culture medium at the indicated concentrations. D1 peptide, which lacks LAR dimerization activity (Fig. 1*D*), was used as a control. D1 did not show a concentration-dependent effect on neurite elongation. In contrast, D1L and D1L-PEG1 treatments resulted in concentration-dependent increases in neurite length. L6 also promoted neurite elongation over a broad concentration range; however, its effect was not strictly concentration-dependent (Fig. 4). L6 at 100 nM, 1 μM, and 3 μM increased neurite length by 1.2–1.3-fold compared with D1-treated control. Similarly, D1L at 1 μM and 3 μM, and D1L-PEG1 at 3 μM increased neurite length by approximately 1.2-fold (Fig. 4). We next examined whether combined treatment with L6 and D1L-PEG1 further enhances neurite elongation. Coapplication of L6 and D1L-PEG1 increased neurite length (∼1.2-fold) at 10 nM and 1 μM; however, it did not enhance neurite elongation beyond the levels observed with individual peptides. These results suggest that both monomeric and dimeric peptides promote neurite extension. However, the enhanced LAR dimerization activity of D1L-PEG1 was not accompanied by a correspondingly greater neurite outgrowth response under the present experimental conditions.

### L6 activated Erk signaling in N2A cells

**Figure 5:**
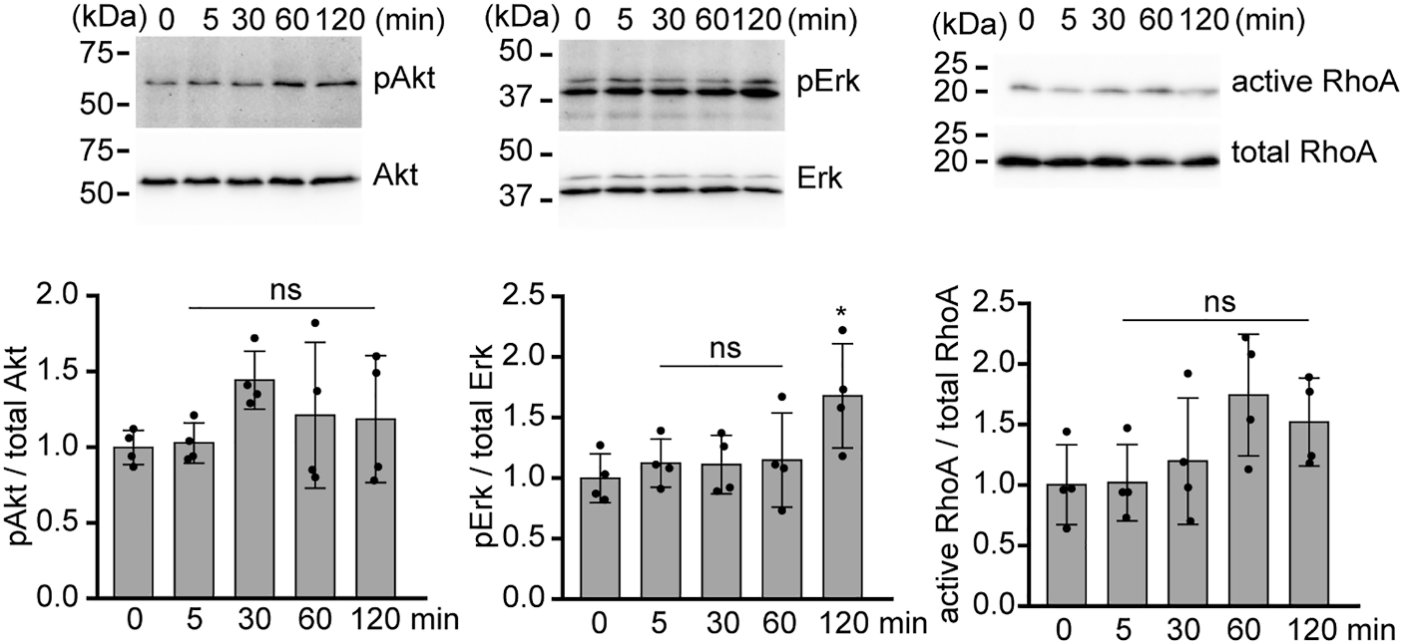
**L6 increases Erk1/2 phosphorylation.** N2A cells transfected with LAR were treated with 10 μM L6 for the indicated times. Levels of phosphorylated Akt (Ser473) and Erk1/2 (Thr202/Tyr204), and GTP-bound active RhoA were analyzed by western blotting or RhoA pull-down assays. L6 treatment significantly increased Erk phosphorylation at 120 min (∼1.7-fold; *p* = 0.023) compared with the 0 min control. Active RhoA showed a modest increase at 60 min (∼1.2-fold; *p* = 0.074). Data are represented as mean ± SD. Individual data points are shown as *circles*. n = 4 independent experiments. ∗*p* < 0.05, ns: not significant; Dunnett’s test. LAR, leukocyte common antigen-related protein.

Several intracellular signaling pathways, including PI3K/Akt, MAPK/Erk, and RhoA, regulate axon growth (37, 38, 39). Interactions between LAR and CSPGs reduce the levels of phosphorylated Akt (Ser473) and Erk1/2 (Thr202/Tyr204), and increase GTP-bound active RhoA in N2A cells and cultured cerebellar granule neurons (9, 19). In contrast, the interaction of LAR with L59 peptide enhanced Akt and Erk1/2 phosphorylation in cultured hippocampal neurons (20). To examine whether L6-induced LAR dimerization is associated with downstream signaling changes, we analyzed the activities of Akt, Erk1/2, and RhoA in N2A cells. N2A cells were transfected with LAR. Cells were then treated with 10 μM L6, and the levels of phosphorylated Akt and Erk1/2 were assessed by western blotting, whereas GTP-bound active RhoA was measured using a RhoA-pull-down assay. L6 treatment significantly increased Erk1/2 phosphorylation at 120 min compared with control. In addition, active RhoA showed a modest but nonsignificant increase at 60 min (Fig. 5*A*). These results suggest that L6 treatment, which promotes LAR dimerization, is associated with the activation of the MAPK/Erk pathway.

## Discussion

LAR plays essential roles in signal transduction in biological processes such as cytoskeletal organization, cell differentiation, adhesion, migration, axon growth, and regenerative neurite outgrowth (6, 7, 9, 11, 13, 14, 15, 40). The phosphatase activity of LAR, which is required for proper signal transduction, is known to be regulated by numerous mechanisms, including oligomerization and ligand binding (19, 22). In the nervous system, monomeric LAR is thought to show phosphatase activity and inhibit axon growth and regeneration, while dimeric or oligomeric LAR has the opposite effect. However, how these forms regulate intracellular signaling related to axon growth and nerve regeneration remains to be fully understood due to the lack of tools for controlling LAR oligomerization states selectively. In this study, we have identified peptides consisting of 14 to 26 amino acids that bound to mLAR-Ig using the RaPID system. Macrocyclic peptide L6 and linear peptide D1L, which were the focus of the present study, showed high affinity for mouse and human LAR-Ig and enhanced LAR dimerization. In addition, L6 and D1L promoted neurite outgrowth in cultured hippocampal neurons. These results suggest that L6 and D1L can be used to explore how monomeric, dimeric, or oligomeric LAR regulates biological processes by controlling its oligomerization state.

Many studies demonstrate that thioether-closed macrocyclic peptides obtained by the RaPID system generally exhibit remarkably high affinity and specificity (27). Interaction affinities of mLAR-Ig with identified peptides in SPR assays were high, with *K*_D_ values of 1.8 to 230 nM. Especially, cyclic peptide L6 and linear peptide D1L exhibited high affinities to mLAR (*K*_D_ = 1.8 nM for L6 and 5.9 nM for D1L). These values were comparable to those determined for the affinity between the target protein and macrocyclic peptide against it, such as human Plexin B1 and a macrocyclic peptide PB1m6 (*K*_D_ = 3.5 nM) (41). Moreover, these values were comparable to or lower than those determined for the affinity between LAR-RPTPs and regulators of their oligomerization, such as *Drosophila* LAR and HSPGs (*K*_D_ = 13 nM for syndecan; 8 nM for dallylike protein), human LAR and heparin (*K*_D_ = 62 nM), or human PTPσ and HSPGs (glypican2 and neurocan; *K*_D_ = 10–20 nM) (18, 23, 42). This indicated that the RaPID system worked well in the present study.

The split luciferase complementary assay is widely used to monitor protein dimerization or oligomerization in living cells. Prior studies using LAR-RPTPs fused with split luciferase fragments at the C terminus demonstrated that an increase in luminescence reflects the promotion of LAR-RPTP dimerization in their endogenous form (43, 44). Therefore, we assessed the ability of the identified peptides to promote mouse and human LAR dimerization using this assay and demonstrated that L6 and D1L could effectively promote both mouse and human LAR dimerization in their endogenous form. Consistent with the high sequence identity (98%, calculated using blastp) of the Ig domain regions between mouse and human LAR, L6 and D1L promoted dimerization of both receptors in the same manner. LAR has four splice variants involving meA and meB (10, 31, 32). The split luciferase complementary assay revealed that L6 interacted with all LAR splice variants, whereas D1L selectively bound to variants containing meA. These results suggest that D1L binds to the region around meA, while L6 binds to a distinct region of LAR. Interestingly, although the other members of type IIa RPTP, PTPσ, and PTPδ, have similar domain structures, and their sequence similarities/identities of predicted L6-and D1L-binding regions are high, these peptides could not promote their dimerization. These results suggest that the nonconserved residues specific to LAR may contact these peptides at the interface of the LAR-peptide complex. Structural predictions further support this interpretation and suggest a mechanistic model in which D1L recognizes the meA insertion region, thereby conferring splice variant selectivity, whereas L6 interacts with a distinct Ig-like domain, enabling broader binding across variants. Notably, the predicted binding interfaces within the Ig-like domain vary across prediction methods, underscoring uncertainty in the precise interaction mode. Therefore, further structural studies will be required to validate these models.

LAR-RPTPs can form a homodimer *via* the ectodomain and/or cytoplasmic domain in living cells (30, 45). By binding to LAR, L6, and D1L peptides may modulate LAR dimerization. D1L likely forms a covalent linkage with LAR *via* its N-terminal chloroacetyl moiety, as supported by the observed decrease in N-terminally acetylated D1L upon induction of LAR dimerization. However, N-terminally chloroacetylated D3 did not exhibit LAR dimerization activity, suggesting that covalent reactivity alone is insufficient. Rather, the specific amino acid sequence of D1L is required for productive interaction with LAR, while covalent linkage likely enhances the stability of this interaction. In addition, we assessed the specificity and potential nonspecific effects of these peptides. FGFR1 and SynCAM1 were selected as representative neuronal receptors containing extracellular Ig domains to assess potential off-target effects of the peptides. Both receptors have established roles in axon growth and neuronal development (46, 47). While L6 showed no detectable effects on the tested FGFR1 and SynCAM1, D1L exhibited limited effects, which were likely mediated by its N-terminal chloroacetyl moiety. Consistent with this, such effects were not observed with Ac-D1L. Furthermore, no apparent peptide aggregation was observed by SDS-PAGE at the concentration used in the split luciferase assays, and no cytotoxicity was observed. Although off-target effects cannot be completely excluded due to the limited number of receptors tested, these findings support the interpretation that the observed LAR dimerization effects are largely attributable to specific peptide–receptor interactions rather than nonspecific peptide aggregation or toxicity.

Dimeric L6 and D1L peptides (L6-PEG5/PEG11 and D1L-PEG1/5/11) showed improved LAR dimerization activities at about 10-fold lower concentration than monomeric ones, as previously reported for dimeric macrocyclic peptide (PB1d6P10 against PlxnB1) (36). Notably, the enhancement was more pronounced for D1L-derived dimers, particularly D1L-PEG1, whereas L6-derived dimers exhibited more modest effects that were dependent on linker length. Multiple residues of previously known macrocyclic peptides often contribute to the interaction with their target proteins (28, 41). Therefore, the presence of dimeric peptides may lead to a 1:2 clamping of two LAR monomers, rather than promote a dimer of LAR dimer although we cannot exclude the possibility that a monomeric peptide may be flanked by two LAR molecules. It should be noted that the activity of the dimeric peptide of both L6 and D1L levels off or plateaus at a high concentration like 10 μM. This was observed due to either the complete saturation of the binding site or an unfavorable dimerization/multimerization event occurred by binding of the dimeric peptide (26). Further structural analysis of the peptide-LAR complex may reveal the stoichiometry.

As some groups have reported the importance of LAR-RPTPs dimerization or oligomerization in neurite elongation (18, 48, 49), we examined whether L6, D1L, dimeric D1L-PEG1, or a combination of L6 and D1L-PEG1 promotes functional dimerization of LAR in cultured hippocampal neurons. Consistent with findings from other groups, treatment with these peptides enhanced neurite elongation compared with D1-treated control. These findings further support a functional link between LAR dimerization/oligomerization and neurite elongation. In addition, L6 and D1L can be used to investigate how intracellular signaling changes when LAR is dimerized or oligomerized.

However, the extent of neurite elongation appeared to be modest. Solid phase binding assays have shown that HS, which contains many saccharide units, induces tetrameric clustering of PTPσ (Coles *et al.*, 2011). Therefore, since sufficient oligomerization of LAR may be required to promote neurite elongation strongly, peptides used in this study might not induce a level of oligomerization high enough to produce a robust effect. Although higher peptide concentrations might enhance this effect, further analysis was precluded by cytotoxicity at elevated concentrations (Fig. S5*D*). Another possible explanation is the heterogeneity of LAR splice variants expressed in cultured hippocampal neurons. Microexon-related LAR-RPTPs splice variants exhibit region- and age-specific expression patterns (31, 50). LAR lacking meA insert is expressed in most hippocampal neurons in adult mice (31) and in a substantial fraction of neurons in juvenile mice (32). Because D1L preferentially binds to meA-containing variants, the coexistence of multiple splice variants may dilute the overall effect on neurite elongation. Consistent with this interpretation, although D1L-PEG1 showed enhanced LAR dimerization activity compared with D1L in the luciferase complementary assay, both monomeric and dimeric D1L peptides produced comparable effects on neurite elongation. These findings underscore the need for further studies to define the specificity of L6-and D1L-mediated neurite elongation, such as through genetic perturbation of LAR or the use of peptide variants with reduced LAR-binding activity. A further possible explanation for the limited effect is the binding heterogeneity of L6 or D1L to LAR. So far, we have not succeeded in making uniform crystals of the LAR-L6 complex. Instead, a few distinct crystal forms have been obtained (data not shown), suggesting that there may be multiple binding sites of L6 on LAR. While some of these L6-LAR interfaces may promote dimerization, others might interfere with it. Such heterogeneity may limit the efficiency of productive receptor clustering. To our knowledge, there are no reports of macrocyclic peptides binding to multiple sites on a single target protein, making this observation potentially noteworthy. These results raise the possibility that not only the extent but also the geometry of LAR dimerization influences downstream signaling. Our result will encourage the determination of the precise binding mode of the LAR-L6 complex.

Treatment with 10 μM peptides did not enhance neurite outgrowth, likely due to cytotoxicity, as noticeable neuronal damage was observed under these conditions (Fig. S5*A*). Although peptide aggregation was considered as a possible cause, neither visible precipitation nor SDS-PAGE evidence of aggregation was detected at 10 μM (Fig. S5, *B* and *C*), suggesting that aggregation is unlikely to account for the observed effect at 10 μM.

In the present study, L6 stimulation induced a significant increase in Erk1/2 phosphorylation in N2A cells, suggesting activation of MAPK signaling pathways associated with neurite outgrowth. This observation is consistent with prior reports showing that activation of LAR signaling by peptides such as L59 enhances Erk and Akt phosphorylation, whereas interactions between LAR and CSPGs suppress these pathways and activate RhoA (9, 19, 20). Because L6 promotes LAR dimerization, the observed activation of Erk signaling suggests that receptor dimerization may be linked to downstream signaling activation. In contrast, only modest and statistically nonsignificant changes were observed in Akt phosphorylation and RhoA activity, indicating that the Erk pathway may be more prominently regulated under the present experimental conditions. However, whether LAR dimerization in the absence of peptide stimulation elicits similar signaling responses remains to be determined.

LAR has attracted much attention as a potential target for treating diabetes, cancer, and neurological disorders (2, 5). Due to the amino acid sequence similarity among LAR-RPTPs, several regulators and inhibitors of LAR have been reported so far. Illudalic acid, isolated from the Basidiomycete *Clitocybe illudens*, its analogs, and the synthesized compound ZINC71414996 bind to the active site of LAR and inhibit phosphatase activity (51, 52, 53). A wedge peptide, consisting of 24 residues derived from the wedge helix-loop-helix domain located between the transmembrane and D1 domain (Fig. 1*A*), undergoes homophilic binding to the LAR wedge domain and stimulates neurite extension in PC12 cells and cultured DRG neurons in the presence of CSPG (9, 54). These regulators and inhibitors act on the cytoplasmic region of LAR. To date, the only known regulators acting on the extracellular region of LAR are LARFN5C and L59 (14). LARFN5C and L59 homophilically bind to FN domains and promote neurite outgrowth in cultured hippocampal neurons and DRG explants (9, 14, 20). Since macrocyclic peptides can be produced in large quantities through chemical synthesis, can be easily modified, and remain stable under physiological reducing conditions (27, 28), our identified L6 macrocyclic peptide should serve not only as a new tool for investigating LAR function but also as a potential therapeutic. However, peptide-based molecules generally face several limitations for *in vivo* application, including limited serum stability, inefficient tissue delivery, and poor penetration across cell membranes and/or the blood-brain barrier (BBB) (55). In particular, BBB permeability remains a critical challenge for targeting the central nervous system. Although BBB permeability has not yet been quantitatively evaluated in this study, our preliminary observations suggest that L6 exhibits limited ability to cross the BBB. Therefore, direct intracerebral administration or the development of BBB-penetrating delivery systems may be required for central nervous system applications. To overcome these limitations, further optimization of pharmacokinetic and delivery properties will be necessary. Potential strategies include incorporation of N-methylation, fluorinated phenylalanine, or D-amino acids, as well as nanoparticle-based delivery systems (56, 57, 58, 59). Future studies will be necessary to evaluate the *in vivo* stability, biodistribution, BBB permeability, and therapeutic efficacy of these peptides.

## Experimental procedures

### Animal

*Fezf2*-EGFP (Stock Tg (Fezf2-EGFP) CO61Gsat/Mmnc; RRID: MMRRC_000293-UNC) and *Fezf2*-tdTomato (Stock Tg (Fezf2-tdTomato) Sz89Gsat/Mmucd; RRID: MMRRC_036540-UCD) mice were obtained from the Mutant Mouse Regional Resource Center (MMRRC), a National Center for Research Resources-National Institutes of Health-funded strain repository, and had been donated to the MMRRC by the National Institute of Neurological Disorders and Stroke-funded GENSAT BAC transgenic project. The day of confirmation of a vaginal plug was designated as embryonic day 0.5 (E0.5). All experiments were conducted in compliance with the guidelines for the use of laboratory animals of The University of Osaka and approved by the Animal Research Committee of The University of Osaka.

### Construction of expression vectors

The coding sequence of Avi-tag and 6×His tag (GLNDIFEAQKIEWHE-GRTK-HHHHHH) was amplified by PCR with primers, 5′–AGTTCTAGAGCGGCCGCTGGAGGTTCCGGTGGTTCCGGTCTGAATG–3′ and 5′–TCTAGAGTCGCGGTCATTAGTGATGGTGATGGTGGTGCTTGGTACG–3′ using pHL-Avi-tag3 as a template and cloned into the NotI site of pEBMulti-neo using the In-fusion HD cloning Plus kit (Clontech) to yield pEBMulti-Avi-tag-6×His, in which the NotI site immediately after the His tag sequence was deleted. The coding sequence of mouse LAR(meA) Ig-like domains was amplified by PCR with primers, 5′–AATAGGTACCGCCACCATGGCTCCCGAGCCAGCCCCAG–3′ and 5′–AATAGCGGCCGCCAGAGCTTTTACTGTGACCTGGGCCGTGGCTTC–3′ using pCAGGS-LAR(meA)-HA (44) as a template and cloned into the KpnI-NotI sites of pEBMulti-Avi-tag-6×His to yield pEBMulti-LAR(meA) Ig×3-Avi-tag-6×His. The coding sequences of mouse LAR(meACD) lacking signal peptide were ligated with the coding sequence of human Igκ signal peptide by PCR using pCAGGS-LAR-HA (44) as a template, and cloned into the SgfI-EcoRI sites of pFC34K-LgBiT TK-neo Flexi Vector or pFC36K-SmBiT TK-neo Flexi Vector (Promega) to yield pFC34K-Igκ-LAR(meA)-LgBiT and pFC36K-Igκ-LAR(meA)-SmBiT, respectively. Coding sequences of LAR(meAB), LAR(meB), and LAR(−) with human Igκ signal peptide were generated by PCR-based mutagenesis using pFC34K-Igκ-LAR(meA)-LgBiT as a template and cloned into pFC34K-LgBiT TK-neo Flexi Vector or pFC36K-SmBiT TK-neo Flexi Vector to yield pFC34K-Igκ-LAR(meAB)-LgBiT, pFC36K-Igκ-LAR(meAB)-SmBiT, pFC34K-Igκ-LAR(meB)-LgBiT, pFC36K-Igκ-LAR(meB)-SmBiT, pFC34K-Igκ-LAR(−)-LgBiT, and pFC36K-Igκ-LAR(−)-SmBiT, respectively. The coding sequences of mouse PTPσ(meB) and PTPδ(meAB) lacking signal peptide were ligated with the coding sequence of human Igκ signal peptide by PCR with primers, 5′–ATCGCGATCGCATGGAGACAGACACACTCCTGCTATGGGTACTGCTGCTCTGGGTTCCAGGTTCCACTGGTGACGAAGAACCACCCAGGTTTATCAGAG–3′ and 5′–GCTGTTTAAACTGTTGCATAATGATCAAAACTGCC–3′ for PTPσ and 5′–ATCGCGATCGCATGGAGACAGACACACTCCTGCTATGGGTACTGCTGCTCTGGGTTCCAGGTTCCACTGGTGACGAGACACCCCCCAGGTTTACAC–3′ and 5′–GCTGTTTAAACGAATTCCGTTGCATAGTGATCAAAG–3′ for PTPδ using cDNA prepared from ICR adult mouse brain as a template, and cloned into the SgfI-EcoRI sites of pFC34K-LgBiT TK-neo Flexi Vector or pFC36K-SmBiT TK-neo Flexi Vector (Promega) to yield pFC34K-PTPσ-LgBiT, pFC36K-PTPσ-SmBiT, pFC34K-PTPδ-LgBiT and pFC36K-PTPδ-SmBiT, respectively.

HSV-TK promoter was amplified by PCR with primers, 5′–CTGGTCGAAATGAGTCTTCGGACCTCGCGGG–3′ and 5′–AGCAGATCTGGTGGCTTTACCAACAGTACC–3′ using LgBiT-PRKAR2A (Promega) as a template, and cloned into the SalI-BglII sites of pCAGGS-5MCS (60) to yield pHSV-TK-5MCS. The coding sequences of LgBiT or SmBiT were amplified by PCR with primers, 5′–CGCGAATTCTGGCAGTTCAGGTGGTGGCG–3′ and 5′–TCACTCGAGTTAGCTGTTGATGGTTACTCGGAAC–3′ for LgBiT and 5′–CGCGAATTCTGGCAGTTCAGGTGGTGGCG–3′ and 5′–GAGGATATCTCTAGATTACAGAATCTCCTCGAACAGCC–3′ for SmBiT using pFC34K-LgBiT TK-neo Flexi Vector or pFC36K-SmBiT TK-neo Flexi Vector as templates, and cloned into the EcoRI-XhoI or EcoRI-EcoRV sites of pHSV-TK-5MCS to yield pHSV-TK-LgBiT or pHSV-TK-SmBiT, respectively. The entire coding sequence of human LAR lacking mini-exons (me) (hLAR(−)) was amplified by PCR with primers, 5′–TTGGGTACCAGATCTATGGCCCCTGAGCCAGCCCCAGGGA–3′ and 5′– TTCGATATCCAATTGCGTTGCATAGTGGTCAAAGCTGCCG–3′ using cDNA prepared from HEK293T cells (293T(ATCC CRL-3216); ATCC), and cloned into KpnI-EcoRV sites of pBluescript-KS(+) to yield pBS-hLAR(−). After replacing signal peptide of hLAR(−) with that of human Igκ to produce Igκ-hLAR(−), the coding sequence of Igκ-hLAR(−) was cloned into BglII-EcoRI sites of pHSV-TK-LgBiT and pHSV-TK-SmBiT to yield pHSV-TK-Igκ-hLAR(−)-LgBiT and pHSV-TK-Igκ-hLAR(−)-SmBiT, respectively. The coding sequence of Igκ-hLAR, which contains meA, C, and D, and lacks the fourth, sixth, and seventh FN, was generated by PCR-based mutagenesis using pHSV-TK-Igκ-hLAR-LgBiT as a template. The entire coding sequences of mouse FGFR1 and SynCAM1 were amplified by PCR with primers, 5′–TCTGCGGCCGCATGTGGGGCTGGAAGTGCCTCCTC–3′ and 5′–CCAGAATTCGGGCGCCGTTTGAGTCCACTGTTGGC–3′ for FGFR1 and 5′–ACCAGATCTGCGGCCGCATGGCGAGTGCTGTGCTG–3′ and 5′–TGAACTGCCAGAATTCGGGATGAAGTACTCTTTCTTTTCTTCG–3′ for SynCAM1 using cDNA prepared from ICR adult mouse brain as a template, and cloned into NotI-EcoRI sites of pHSV-TK-LgBiT and pHSV-TK-SmBiT by ligation or In-fusion reaction (Clontech) to yield pHSV-TK-FGFR1-LgBiT, pHSV-TK-FGFR1-SmBiT, pHSV-TK-SynCAM1-LgBiT, and pHSV-TK-SynCAM1-SmBiT, respectively. A Rho binding domain (RBD) of mouse Rhotekin was amplified by PCR with primers, 5′–CTGGGATCCATCCTGGAGGACCTCAATATGC–3′ and 5′–CGAGTCGACCTAGCCTGTCTTCTCCAGCACCT–3′ using cDNA prepared from ICR adult mouse brain as a template, and cloned into BamHI-SalI sites of pGEX-6P-1(GE Healthcare) to yield pGEX-Rhotekin-RBD.

### Preparation of recombinant proteins

Soluble recombinant LAR(meA)-Ig×3-Avi-tag-6×His was prepared as described in a prior report (61). In brief, after adding biotin (100 μM) to the culture medium, plasmids encoding LAR(meA)-Ig×3-Avi-tag-6×His and pDisplay-BirA-ER (a gift from Alice Ting; Addgene plasmid #20856) (62) were cotransfected into FreeStyle 293F cells using 293fectin (Thermo Fisher Scientific) according to the manufacturer’s instructions. Seventy-two hours after transfection, cell-conditioned media were harvested, and biotinylated recombinant proteins were purified by Talon metal affinity resin (Clontech). Before elution by imidazole (150 mM) and dialysis against PBS, peptide N-glycosidase F enzyme was added for deglycosylation of recombinant LAR(meA)-Ig×3-Avi-tag-6×His.

Recombinant GST-Rhotekin-RBD was prepared as described in a prior report with minor modifications (63). *E. coli* BL21(DE3) cells transformed with pGEX-Rhotekin-RBD were cultured in LB medium containing ampicillin at 30 °C with shaking until OD_600_ reached 0.7. Protein expression was induced by the addition of 0.5 mM IPTG, followed by incubation at 20 °C with shaking for 16 h. Cells were harvested by centrifugation at 4000*g* for 20 min at 4 °C, resuspended in 10 ml ice-cold GST buffer (25 mM Tris-HCl, 1 M NaCl, 10 mM DTT, 200 μg/ml lysozyme, 0.1% Triton X-100, protease inhibitor cocktail (Nacalai Tesque)), and lysed by sonication on ice. Cell lysates were clarified by centrifugation at 15,000*g* for 1 h at 4 °C.

### Screening of anti-LAR peptides

Thioether macrocycles targeting mouse LAR extracellular Ig-like domains were screened using RaPID system as described in a prior report (64) with modifications. Briefly, the mRNA NNK library was ligated with a puromycin linker by T4 ligase. Transfer RNAs (tRNAs) aminoacylation was performed in the reactions containing 40 mM tRNA^fMet^_CAU_ and flexizyme aminoacylation ribozyme, 600 mM MgCl_2_, 5 mM each activated noncanonical amino acid (*N*-chloroacetyl-L-Tyr-CME, *N*-chloroacetyl-D-Tyr-CME) and 100 mM Hepes-KOH (pH 7.5) on ice for 2 h followed by the ethanol precipitation purification (65).

The resulting mRNA-puromycin library was translated in a methionine-deficient flexible *in vitro* translation system at a scale of 150 μl total volume in the presence of 50 μM ClAc-L-Tyr-tRNA^fMet^_CAU_ or ClAc-D-Tyr-tRNA^fMet^_CAU_ for 30 min at 37 °C. After a 12-min incubation at 25 °C, then 30 μl of EDTA (200 mM, pH 8.0) was added to the mixture followed by a 37 °C 30 min incubation to promote macrocyclization. The fused macrocycle–mRNA library was subsequently reverse transcribed by M-MLV reverse transcriptase RNase H minus (M-MLV RT [H-], Promega) for 1 h at 42 °C with the addition of the reverse transcription buffer. The mixture was then combined with 282.5 μl of blocking buffer (Tris-buffered saline with Tween 20 containing 0.2% acetylated bovine serum albumin; Life Technologies) and then incubated with mLAR-Ig immobilized on Dynabeads for 30 min at 4 °C for affinity selection. The resultant complementary DNAs were eluted from the beads by heating at 95 °C for 5 min. The fractional recovered cDNAs were assessed by real-time PCR quantification using SYBR Green I on a LightCycler 2.0 thermal cycler (Roche). The enriched DNA libraries were recovered by PCR and used as a template for the transcription reaction for the next round of selection. For the later round of selections, the procedure was performed as previously, except the translation scale was reduced to 5 μl and the library was first reverse transcribed by M-MLV (H-) and subjected to six serial incubations with Dynabeads M-280 (counter selection, 30 min each at 4 °C) before positive selection with mLAR-Ig immobilized beads to remove bead binders. The amplified cDNAs from the seventh and eighth rounds were subjected to DNA deep sequencing using the MiSeq sequencing system (Illumina). The water solubilities of the selected peptides were analyzed using the online tool CamSol5 at pH 7.0.

### Chemical synthesis of peptides

Monomeric macrocyclic peptides were synthesized by standard Fmoc solid-phase peptide synthesis at the scale of 25 μmol on NovaPEG Rink Amide resin (Merck Millipore) using a Syro Wave automated peptide synthesizer (Biotage) as described in a prior report (64). Briefly, the resulting peptide was first subjected to the N-terminal acylation followed by the global deprotection by a solution of 92.5% TFA, 2.5% water, 2.5% triisopropylsilane, and 2.5% ethanedithiol, to yield the free linear peptides with N-terminal acylation (chloroacetylation or acetylation). The peptides were washed and precipitated by diethylether for 5 times and recovered by centrifugation. The resulting pellet was dissolved in 10 ml triethylamine containing DMSO and incubated for 1 h at 25 °C to yield the corresponding macrocycle. The peptide suspensions were then acidified by the addition of TFA to quench the macrocyclization reaction. The macrocycle was purified by reversed-phase high-performance liquid chromatography using a Prominence LC20-AP system (Shimadzu) with a Chromolith Prep Column (100 mm by 25 mm internal diameter; Merck Millipore), and the elution was performed with a linear gradient condition from 0.1% TFA in water to 0.1% TFA in acetonitrile. Purified peptides were lyophilized *in vacuo* and molecular mass was confirmed by MALDI-TOF MS (AutoFlex II; Bruker Daltonics).

Dimeric macrocyclic peptides were synthesized as described in a prior report (36) with modifications. Briefly, DMF-engorged NovaPEG Rink Amide resins were first coupled twice with 0.5 M *Nα, Nβ*-di-Fmoc-2,3-diaminopropionic acid in the presence of 0.5 M HBTU/HOBt and 0.5 M DIPEA in DMF, followed by three DMF washes, and Fmoc-deprotection with 40% piperidine in DMF. Then Fmoc-NH-PEG_X_-COOH linker (x is 1, 5, or 11) was coupled to the resulting resin similarly. The resulting resin was subjected to the amino acid coupling, N-terminal acylation, and deprotection procedures as described previously. Then the dimeric peptides were cyclized, purified, and confirmed similarly as for the preparation of monomeric peptides.

### SPR analysis of macrocyclic peptides

SPR analysis was performed using a Biacore T200 (GE Healthcare) as described in a prior report (36). Avi-tagged mLAR-Ig was immobilized on the surface of Series S sensor chip SA with a density of approximately 1200 response units. The running buffer was HBS-EP + buffer (10 mM Hepes-KOH, pH 7.4, 150 mM NaCl, 50 μM EDTA, 0.05% Tween 20) containing 0.1% DMSO. A range of five different concentrations of each peptide was injected at 25 °C at a flow rate of 30 μl/min, and a single-cycle kinetics method was employed to quantify the binding affinity and kinetic constants. The binding sensorgrams were fitted to the standard 1:1 binding model with the use of Biacore evaluation software (GE Healthcare).

### Cell culture

HEK293T cells and N2A cells were maintained in Dulbecco’s Modified Eagle’s Medium (Nacalai Tesque) supplemented with 10% fetal bovine serum (Nichirei Biosciences), 100 U/ml penicillin, and 100 μg/ml streptomycin at 37 °C in a humidified 5% CO_2_ atmosphere. FreeStyle 293F cells (Thermo Fisher Scientific) were cultured in FreeStyle 293 expression medium (Thermo Fisher Scientific) at 37 °C in a humidified 8% CO_2_ atmosphere on an orbital shaker platform rotating at 135 rpm.

### Split luciferase assay

A split luciferase assay was conducted using the Nano-Glo live cell assay system (Promega) according to the manufacturer’s instructions. HEK293T cells were cotransfected with expression vectors for LgBiT- and SmBiT-fused mLAR, PTPσ, PTPδ, FGFR1, SynCAM1, or hLAR using polyethyleneimine (PEI Max; Polyscience). After 24 h, HEK293T cells were harvested with 2 mM EDTA in PBS. Cells in 100 μl medium were seeded in each well of a 96-well plate (Corning) and allowed to adhere for 5 to 9 h. Then, the culture medium was exchanged with 80 μl prewarmed Opti-MEM I (Thermo Fisher Scientific), and 20 μl substrate solution was added. After 5 min incubation, luminescence was measured at 1 min intervals (0.5 s exposure) for 20 min measurements 1 to 20) using a microplate reader (GloMax Discover microplate reader; Promega, Centro XS3 LB960; Berthold Technologies, or SpectraMax iD5; Molecular Devices). Subsequently, a macrocyclic peptide in 10 μl of Opti-MEM I was added, and the changes in luminescence were measured for an additional 60 min (measurements 21–80). The ratio of luminescence at the 50th measurement to that at the 20th measurement was calculated as an index of dimerization activity and normalized to the DMSO-treated control.

### Primary neuron culture and neurite elongation assay

Primary hippocampal cultures were prepared from *Fezf2*-EGFP or *Fezf2*-tdTomato mice at E18.5. The tissues were digested with papain (Worthington Biochemical Corporation) in a papain solution (DL-cysteine, 0.2; bovine serum albumin, 0.2; glucose, 5; DNase I (Roche diagnostics), 0.1; MgCl_2_, 0.286 in mg/ml) for 20 min at 37 °C. After washing with PBS, the tissues were triturated and filtered through a 40-μm cell strainer (BD Biosciences). The dissociated neurons were plated on polyethyleneimine-coated 4-well chamber slides (Matsunami Glass Ind) in 25% horse serum, 25% Hank’s balanced salt solution, and 50% Neurobasal medium (Thermo Fisher Scientific) containing 100 U/ml penicillin and 100 μg/ml streptomycin at 5 × 10^4^ cells/well. After 3 h, the medium was exchanged with Neurobasal medium containing B27-supplement (Thermo Fisher Scientific), GlutaMAX-I (Thermo Fisher Scientific), 100 U/ml penicillin, 100 μg/ml streptomycin, and 1 μM macrocyclic peptide. After 48 h of macrocyclic peptide treatment, the neurons were fixed with 4% paraformaldehyde/4% sucrose in PBS and immunostained with chicken anti-GFP (Abcam) or goat anti-RFP (Sickgen) and mouse anti-tau-1(clone PC1C6; Merck Millipore) antibodies, followed by incubation with donkey Alexa Fluor-488 conjugated anti-chicken IgY and Alexa Fluor-568 conjugated anti-mouse IgG (Thermo Fisher Scientific), or donkey Alexa Fluor-488 conjugated anti-mouse IgG and Alexa Fluor-568 conjugated anti-goat IgG (Thermo Fisher Scientific). Images of Fezf2 promoter-driven fluorescence-positive neurons were acquired with a fluorescence microscope (BZ X-1000; Keyence) using the Plan Apochromat 40× lens. The longest neurite was measured using the ImageJ plugin NeuronJ (66). Images were collected from three independent experiments.

### Western blot analysis

To examine the signaling effects of L6 peptide, N2A cells seeded in 6-well plates were transfected with pCAG-LAR-V5 (44). Two days after transfection, cells were stimulated with 10 μM L6 for 5, 30, 60, or 120 min. Cells were washed twice with ice-cold PBS and lysed in 300 μl lysis buffer (50 mM Tris-HCl, pH 7.4, 150 mM NaCl, 1 mM EDTA, 1% NP-40) supplemented with protease and phosphatase inhibitor cocktail (Nacalai Tesque or Roche Diagnostics). Cell lysates were clarified by centrifugation at 15,000 rpm for 15 min at 4 °C, and protein concentrations were measured using the Bradford assay or the Qubit 4 fluorometer (Thermo Fisher Scientific). For the detection of active RhoA, GST-Rhotekin-RBD was coupled to glutathione magnetic beads (Sigma-Aldrich). Cell lysates were incubated with the beads for 2 to 3 h at 4 °C, and bound proteins were eluted with Laemmli SDS sample buffer. Antibodies used were mouse anti-phospho-Akt (Ser473, 587F11), rabbit anti-phospho-Akt (Ser473, D9E), rabbit anti-phospho-ERK1/2 (p44/42, 137F5), rabbit anti-Akt (11E7), rabbit anti-ERK1/2 (137F5), and rabbit anti-RhoA (67B9) (all from Cell Signaling Technology).

### Protein-peptide complex structure prediction

Structure prediction of a complex comprising three Ig-like domains of LAR (LAR-Ig×3) and peptides was performed using ColabFold, HADDOCK, and Rosetta. The structures of LAR-Ig×3, L6, and D1L were initially predicted using ColabFold (ColabFold v1.5.5: AlphaFold2 using MMseqs2 with cyclic offset; (34)). Protein-peptide docking was subsequently carried out using ColabFold, HADDOCK (HADDOCK 2.4 web server; (35)), and Rosetta (Flexpepdock *via* ROSIE server; (33)). The ColabFold-generated complex model was used as a template for Rosetta-based docking.

### Peptide self-dimerization assay

To assess peptide self-dimerization, 1 μg of denatured or non-denatured L6 and D1L peptides as well as denatured L6-PEG1 and D1L-PEG1 peptides were separated by SDS-PAGE using 15% Tris-Tricine gel, and stained with Coomassie Brilliant Blue G-250 (Nacalai Tesque) according to a prior report (67).

### Cell viability assay

Cell viability was assessed using the WST-8 assay (Cell Counting Reagents SF; Nacalai Tesque) according to the manufacturer’s instructions. HEK293T or N2A cells were seeded at 1 × 10^4^ cells/well in 96-well plates. After 24 h, the medium was replaced with 100 μl of prewarmed fresh medium with 10 μl L6 solution or DMSO as a control. Twenty-four hours after peptide treatment, the medium was replaced with 100 μl prewarmed fresh medium, and 10 μl of cell counting solution was added. After incubation for 3 h, absorbance at 450 nm was measured using a microplate reader (SpectraMax iD5; Molecular Devices) with 600 nm as a reference wavelength. Cell viability was calculated using the following equation.

$$\text{Cell}\;\text{viability}\;\left(\%\right)=\frac{\text{Absorbance}\;\text{of}\;\text{sample}-\text{Absorbance}\;\text{of}\;\text{medium}}{\text{Absorbance}\;\text{of}\;\text{control}-\text{Absorbance}\;\text{of}\;\text{medium}}\times 100$$

### Statistical analysis

Statistical analyses were conducted using JMP statistical software (JMP Pro 17; JMP Statistical Discovery). Statistical significance was assessed with Dunnett’s test, Steel’s test, Student’s *t* test, two-way repeated measures ANOVA followed by Dunnett’s *post hoc* test, or two-tailed Jonckheere-Terpstra trend test. *p* < 0.05 was considered statistically significant.

## Data availability

All experimental data that are not contained within the article or the supporting information are available upon request by contacting author Makoto Sato (sato.makoto.med@osaka-u.ac.jp).

## Supporting information

This article contains supporting information.

## Conflict of interest

The authors declare that they have no conflicts of interest with the contents of this article.
