# Supplementary material for: Macrocyclic and linear peptides promote LAR dimerization and neurite outgrowth
Source: J Biol Chem. 2026 Jul 16;302(9):113340. doi: 10.1016/j.jbc.2026.113340 (PMC13486334; doi:10.1016/j.jbc.2026.113340)
Supplement: Supplementary file 1 [file mmc1.pdf]

Supporting information

Macrocyclic and linear peptides promote LAR dimerization and neurite outgrowth

Misato Yasumura, Yuchen Zhang, Tokuichi Iguchi, Naoakusa Fujimura, Kilian Colas, Yuichiro Oka, Hiroyuki Oshikan, Tsuyoshi Inoue, Hiroaki Suga, Makoto Sato

List of materials included.

Figure S1
Figure S2
Figure S3
Figure S4
Figure S5

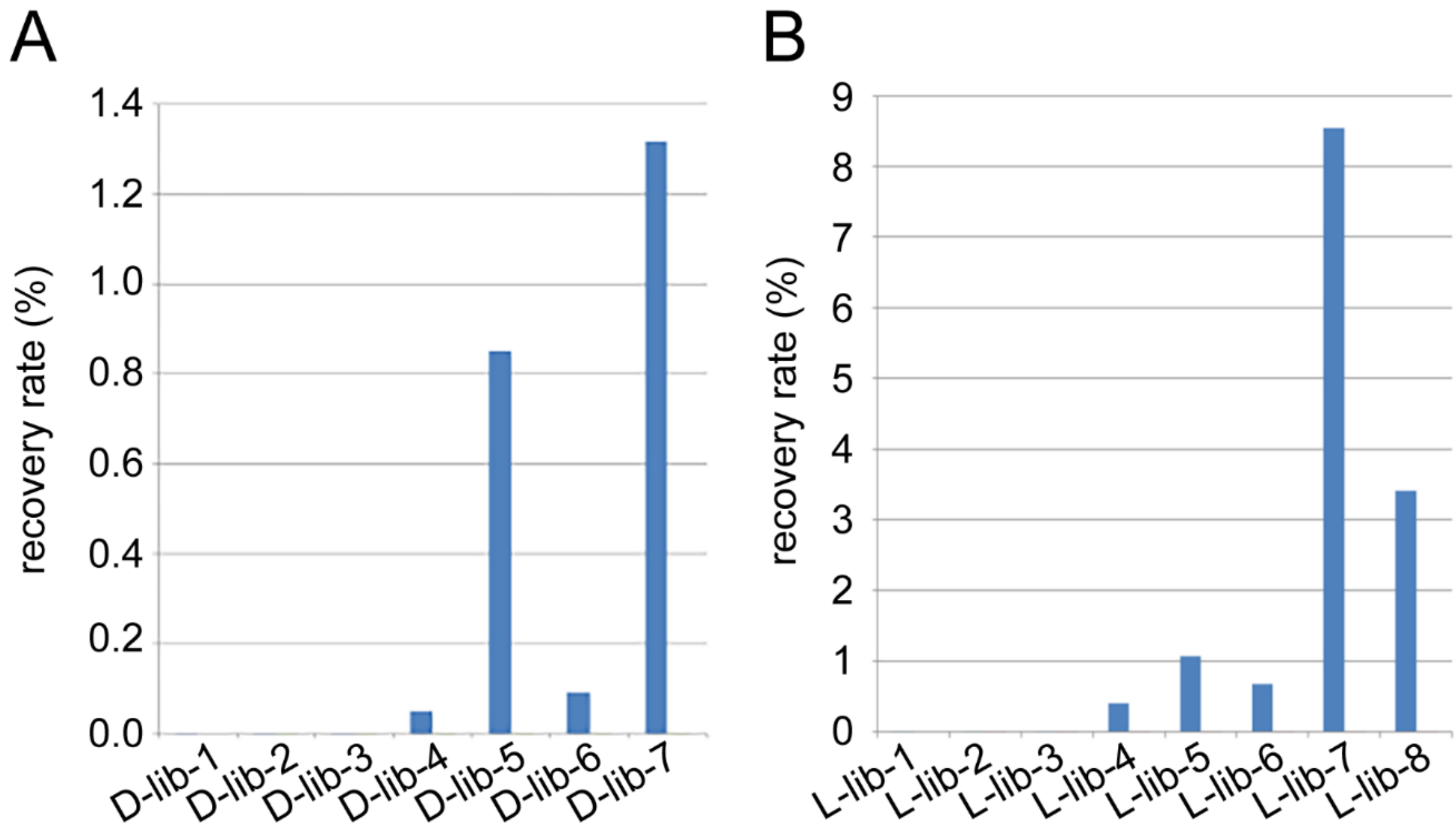

Figure S1. Enrichment of macrocyclic peptides that bind to the extracellular Ig-like domains of mouse LAR (mLAR-Ig) with the use of the RaPID system.

Recovery rates for cDNAs eluted from mRNA-linked macrocyclic peptides of the (A) ClAc-D-Tyr-initiated library (D-lib) and (B) ClAc-L-Tyr-initiated library (L-lib) that bound to mLAR-Ig. The recovery rates reached a plateau after the 7th or 8th round of selection.

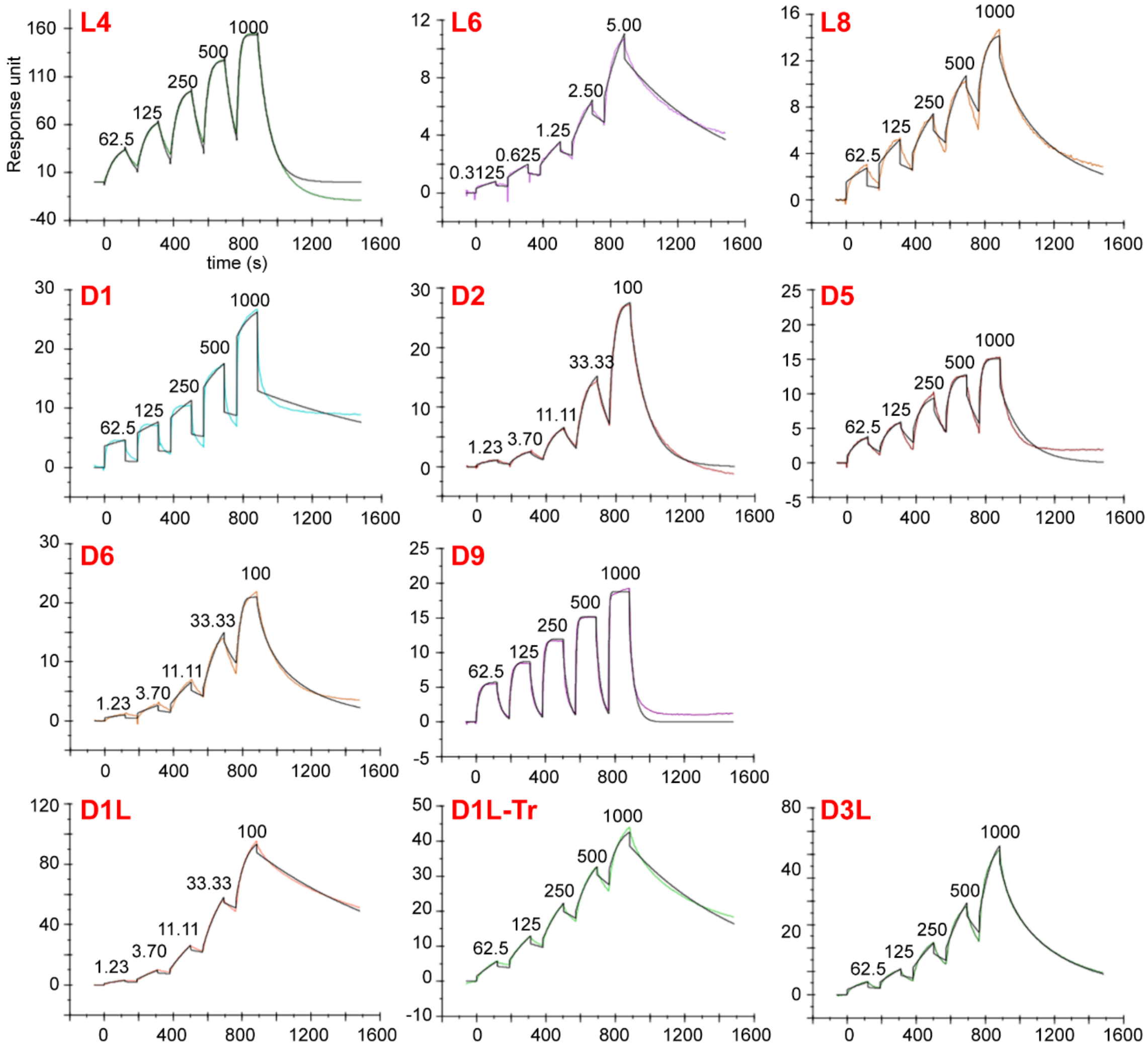

Figure S2. Binding kinetics for macrocyclic peptides and mLAR-Ig.

The binding of the indicated macrocyclic peptides to mLAR-Ig was characterized by SPR analysis. Fitted curves are shown in black, while measured curves are shown in other colors.

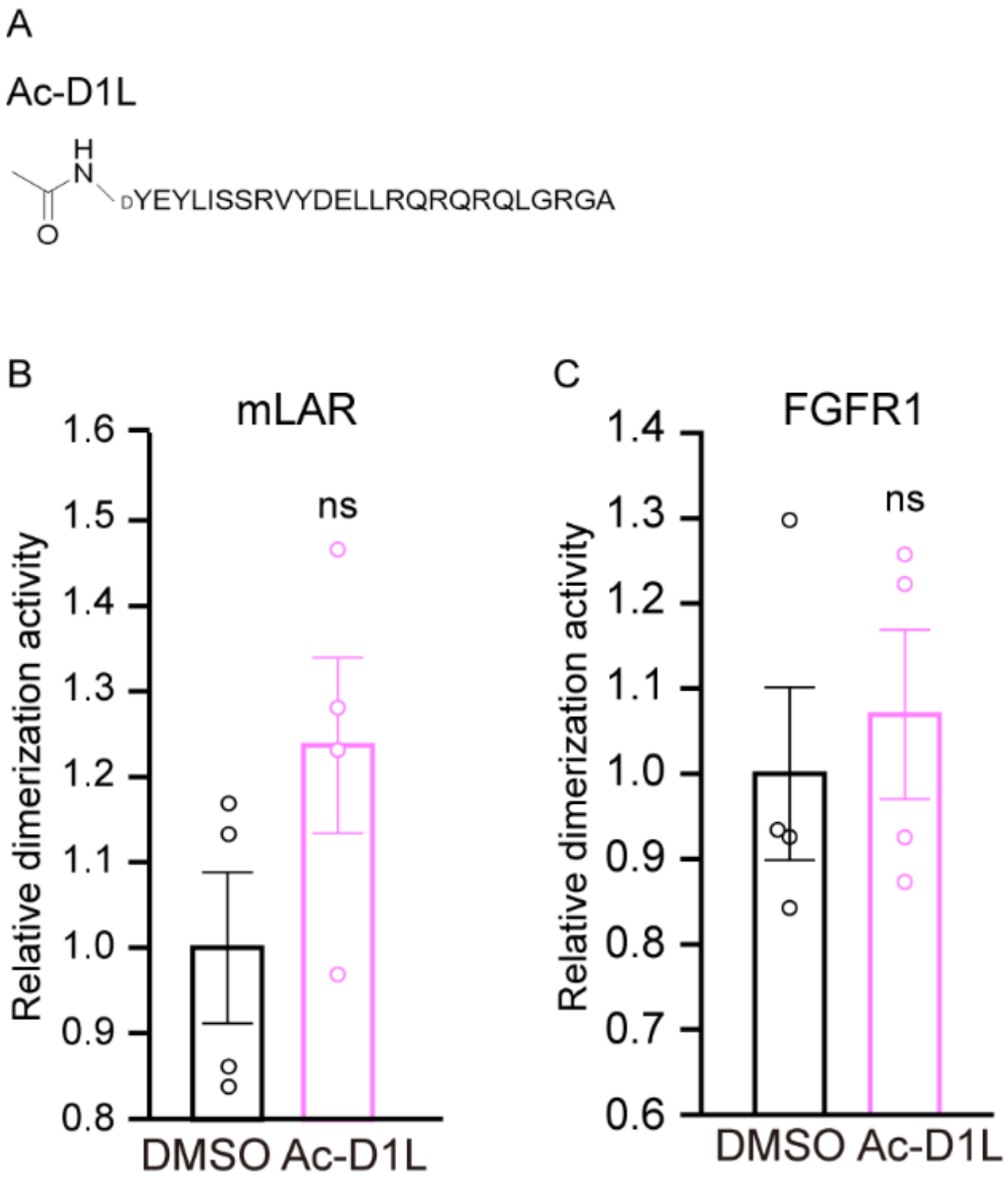

Fig. S3 Effects of N-terminally acetylated D1L (Ac-D1L) on LAR and FGFR1 dimerization.

A. Structure of N-terminally acetylated D1L (Ac-D1L).

B. Effect of Ac-D1L on mLAR dimerization in HEK293T cells co-expressing mLAR-LgBiT and mLAR-SmBiT. Application of Ac-D1L showed a tendency to enhance LAR dimerization (1.23 ± 0.21; p = 0.10) compared to DMSO-treated control (1.00 ± 0.18); however, this effect did not reach statistical significance. Data are represented as mean ± SD. Individual data points are shown as closed circles. n = 4 independent experiments. Each experiment was performed in triplicate or quadruplicate.

C. An effect of Ac-D1L on FGFR1 dimerization in HEK293T cells co-expressing FGFR1-LgBiT and FGFR1-SmBiT. Application of Ac-D1L did not inhibit FGFR1 dimerization (1.06 ± 0.20; p = 0.61) compared to DMSO-treated control (1.00 ± 0.04). The DMSO-treated control was the same as that in Fig. 2E. n = 4 independent experiments. Each experiment was performed in triplicate or quadruplicate.

All data are represented as mean ± SD. Individual data points are shown as open circles.

ns: not significant; student's t-test.

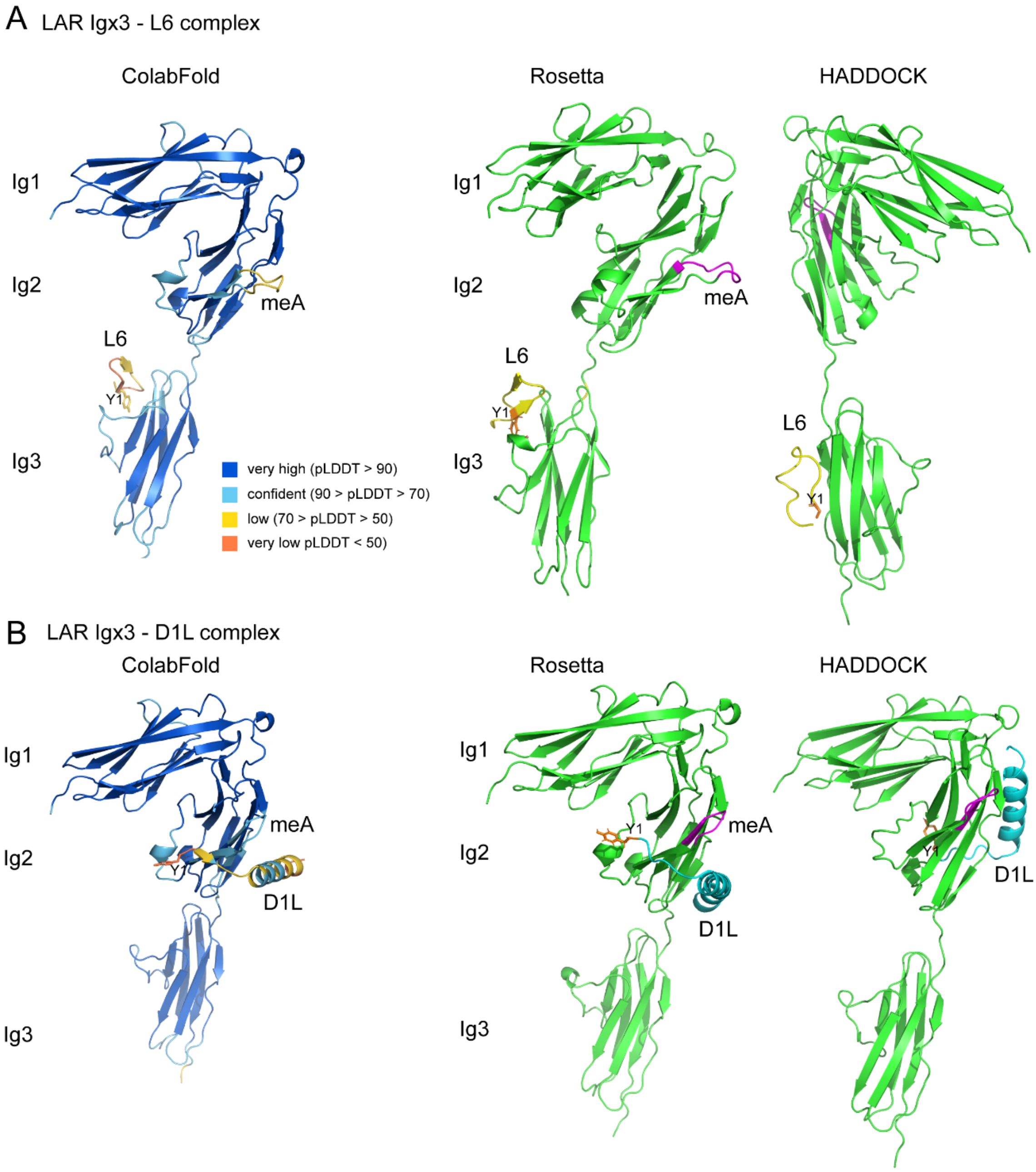

Figure S4. Structural predictions of LAR-Ig-L6 and LAR-Ig-D1L complexes.

The structures of LAR-Ig-L6 (A) and LAR-Ig-D1L (B) complexes were predicted using ColabFold (AlphaFold2 using MMseqs2 with cyclic offset), Rosetta, and HADDOCK based on the amino acid sequences of LAR(meA) Ig×3, L6, and D1L. Structural images were generated using PyMOL. In the ColabFold models, structures are color-coded by pLDDT values, which indicate prediction confidence. Green: LAR Ig domains; magenta: microexon A (meA); yellow: L6; blue: D1L; Y1: the first tyrosine residue of the peptides.

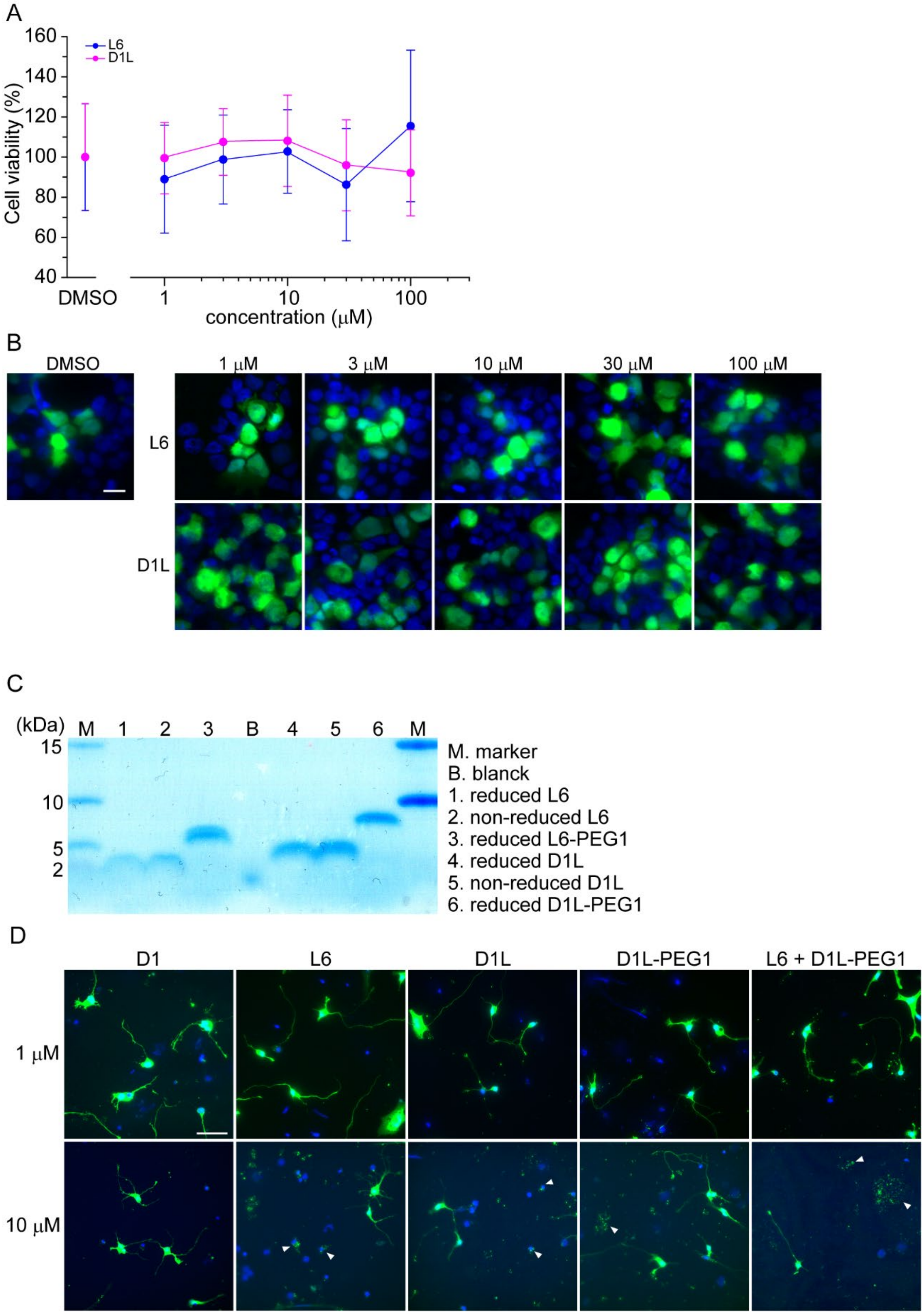
A
L6
D1L
Cell viability (%)
160
140
120
100
80
60
40
DMSO
1
10
100
concentration (μM)
B
DMSO
1 μM
3 μM
10 μM
30 μM
100 μM
L6
D1L
C
(kDa)
M
1
2
3
B
4
5
6
M
15
10
5
2
M. marker
B. blanck
1. reduced L6
2. non-reduced L6
3. reduced L6-PEG1
4. reduced D1L
5. non-reduced D1L
6. reduced D1L-PEG1
D
D1
L6
D1L
D1L-PEG1
L6 + D1L-PEG1
1 μM
10 μM

Figure S5. Cytotoxicity and self-assembly of L6 and D1L.

A. Cell viability of HEK293T cells was measured using the WST-8 assay. Treatment with either L6 or D1L for 18 h did not affect cell viability. Data are represented as mean ± SD (n = 4 independent experiments).

B. Effect of L6 or D1L on HEK293T cell morphology. GFP-transfected cells were treated with the peptides at the indicated concentrations for 18 hr. Blue, DAPI; green, GFP. Scale bar, 20 μm.

C. CBB staining analysis of L6 and D1L under reducing or non-reducing conditions. One μg of L6 (1.9 kDa) or D1L (3.1 kDa) was loaded and compared with the molecular sizes of dimeric peptides.

D. Cytotoxicity of peptides in cultured hippocampal neurons. A large number of damaged neurons were observed in cultures treated with 10 μM peptides. White arrowheads indicate damaged neurons. Scale bar, 50 μm.
